# Liver cancer stem cell dissemination and metastasis: uncovering the role of NRCAM in hepatocellular carcinoma

**DOI:** 10.1186/s13046-023-02893-w

**Published:** 2023-11-22

**Authors:** Lingyun Zhou, Linye He, Chang-Hai Liu, Huandi Qiu, Li Zheng, Klarke Michael Sample, Qin Wu, Jiaxin Li, Kunlin Xie, Javier Ampuero, Zhihui Li, Duoduo Lv, Miao Liu, Manuel Romero-Gómez, Yiguo Hu, Hong Tang

**Affiliations:** 1 Center of Infectious Diseases, West China Hospital of Sichuan University, 37 GuoXue Lane, Chengdu, 610041 Sichuan Province China; 2 Division of Infectious Diseases, State Key Laboratory of Biotherapy, West China Hospital of Sichuan University, Chengdu, China; 3 Thyroid and Parathyroid Surgery Center, West China Hospital of Sichuan University, Chengdu, China; 4 State Key Laboratory of Biotherapy and Cancer Center, West China Hospital of Sichuan University, 37 GuoXue Lane, Chengdu, 610041 Sichuan Province China; 5 Department of Liver Surgery and Liver Transplantation Centre, West China Hospital of Sichuan University, Chengdu, China; 6 Digestive Diseases Unit, Virgen del Rocío University Hospital, SeLiver Group at Institute of Biomedicine of Seville (IBIS: HUVRocío/CSIC/US), University of Seville, Seville, Spain; 7 Digestive Disease Department and CIBERehd, Virgen del Rocío University Hospital, Avenida Manuel Siurot S/N, 41013 Seville, Spain; 8 National Clinical Research Center for Geriatrics, West China Hospital of Sichuan University, Chengdu, China

**Keywords:** Liver cancer stem cells (LCSCs), Hepatocellular carcinoma (HCC), Metastasis, NRCAM, Single-cell RNA sequencing, Epithelial-mesenchymal transition (EMT), Metastasis-related matrix metalloproteinases (MMPs), MACF1, β-catenin signaling pathway

## Abstract

**Background:**

Liver cancer stem cells (LCSCs) play an important role in hepatocellular carcinoma (HCC), but the mechanisms that link LCSCs to HCC metastasis remain largely unknown. This study aims to reveal the contributions of NRCAM to LCSC function and HCC metastasis, and further explore its mechanism in detail.

**Methods:**

117 HCC and 29 non-HCC patients with focal liver lesions were collected and analyzed to assess the association between NRCAM and HCC metastasis. Single-cell RNA sequencing (scRNA-seq) was used to explore the biological characteristics of cells with high NRCAM expression in metastatic HCC. The role and mechanism of NRCAM in LCSC dissemination and metastasis was explored in vitro and in vivo using MYC-driven LCSC organoids from murine liver cells.

**Results:**

Serum NRCAM is associated with HCC metastasis and poor prognosis. A scRNA-seq analysis identified that NRCAM was highly expressed in LCSCs with MYC activation in metastatic HCC. Moreover, NRCAM facilitated LCSC migration and invasion, which was confirmed in MYC-driven LCSC organoids. The in vivo tumor allografts demonstrated that NRCAM mediated intra-hepatic/lung HCC metastasis by enhancing the ability of LCSCs to escape from tumors into the bloodstream. Nrcam expression inhibition in LCSCs blocked HCC metastasis. Mechanistically, NRCAM activated epithelial-mesenchymal transition (EMT) and metastasis-related matrix metalloproteinases (MMPs) through the MACF1 mediated β-catenin signaling pathway in LCSCs.

**Conclusions:**

LCSCs typified by high NRCAM expression have a strong ability to invade and migrate, which is an important factor leading to HCC metastasis.

**Supplementary Information:**

The online version contains supplementary material available at 10.1186/s13046-023-02893-w.

## Background

Liver cancer is the sixth most diagnosed cancer (fifth for men), yet it ranks second in cancer mortality rate for men. Hepatocellular carcinoma (HCC) is the predominant histological liver cancer subtype globally, accounting for 75–85% of primary cases [1]. The majority of HCC cases are diagnosed at advanced stages, at which point there is a lack of therapies that can extend survival [2]. Moreover, some patients experience rapid progression characterized by early disease recurrence, progression, and metastasis [3].

Early-stage HCC (prior to surgery) can be classified into three broad groups according to their maturation status: 1) hepatocyte-type (a hyper-intense disease with good prognosis), 2) intermediate type, and 3) stem cell-type (hypo-intense). The latter type is associated with poor prognosis, high serum Alpha-fetoprotein (AFP) levels, and *FOXM1* expression (as opposed to HNF4A) [4]. Epithelial-mesenchymal transition (EMT) is a key feature by which cancer cells gain highly migrative and invasive properties. During EMT, cells lose their epithelial characteristics, such as cell–cell adhesion and apical-basal polarity, and acquire mesenchymal traits, including enhanced motility and resistance to apoptosis. This transition empowers cancer cells to breach tissue boundaries, invade surrounding stroma, and ultimately disseminate to distant organs, leading to the formation of metastatic lesions. Moreover, accumulating evidence suggests that EMT is not a binary switch but exists as a spectrum, with cells displaying varying degrees of epithelial and mesenchymal features, contributing to the heterogeneity observed in tumors. Liver cancer stem cells (LCSCs) have emerged as pivotal players in HCC. Their unique properties enable self-renewal and differentiation, driving advanced disease, and are associated with a poor prognosis for HCC patients [4–8]. Additionally, LCSCs, in conjunction with the EMT process, significantly impact HCC metastasis [9]. Understanding the intricate interplay between LCSCs, EMT, and HCC metastasis may be key to developing targeted therapies, promising new avenues to combat this deadly disease.

Wingless-Type MMTV Integration Site Family (WNT)/β-catenin signaling contributes to HCC development and is associated with LCSC self-renewal, invasion, and metastasis [8, 10]. WNT activation promotes EMT through glycogen synthase kinase-3β (GSK3β) inhibition, stabilizing β-catenin and enabling its nuclear translocation, enhancing EMT-associated transcription factors (including LEF and TCF) [8]. Despite strong evidence linking the WNT/β-catenin signaling pathway to HCC, the pathway has proved challenging to target [11]. Therefore, novel targets and biomarkers of WNT/β-catenin signaling could provide prognostic benefits for HCC patients, especially those with stem cell-type disease.

Several cell adhesion molecules have been associated with WNT/β-catenin signaling in recent years [12]. Neuronal cell adhesion molecule (NRCAM) is a member of the L1 family [13] and a target of β-catenin via LEF/TCF binding sites in its promoter [14]. Moreover, NRCAM forms a positive feedback loop by inducing the MAPK/Erk and PI3K/Akt pathways, activating GSK3β/β-catenin [15]. NRCAM is highly expressed in various cancers, including pediatric neuroblastoma [16], melanoma [14], colon [12, 14], pancreatic [17], and thyroid cancer [15]. NRCAM expression also promotes malignant cell transformation, cell motility, and metastatic disease [12, 14, 17].

NRCAM is an interesting potential therapeutic target due to it being a transmembrane protein (and may have high drug bioavailability). NRCAM is also known to be present in serum and is perhaps cleaved from the cytoplasmic membrane by Matrix metalloproteinases (MMPs) [12]. This pattern of cleavage is intriguing because many MMPs are regulated by WNT/β-catenin signaling, which is associated with the progression of various cancers. MMPs constitute a family of enzymes crucial in the tumor microenvironment, and play a fundamental role by degrading extracellular matrix components, facilitating cancer cell invasion and migration. In liver cancer, MMPs are implicated in the breakdown of tissue barriers, enabling cancer cells to infiltrate surrounding tissues and metastasize to distant organs [3]. However, the specific MMPs and mechanism involved in HCC metastasis remain unknown.

For these reasons, the study herein utilizes single-cell RNA sequencing (scRNAseq) and MYC-driven LCSC organoids from murine liver cells to explore the role of NRCAM in LCSC function and HCC metastasis. In addition, serum NRCAM is also examined as a potential biomarker for HCC and metastasis via a cross-sectional study.

## Methods

### Experimental design and sample collection

From March 2017 to December 2021, 146 patients with hepatic lesions were studied at the West China Hospital (Sichuan University, CN). The patients either underwent surgical liver resection or transcatheter arterial chemoembolization (TACE) treatment, and their clinical and histology information were collected from the medical record system. Out of these, 117 patients were confirmed to have HCC through a biopsy following surgical resection; whereas 29 patients were diagnosed with benign lesions (liver cysts, hepatic hemangioma, hepatocellular adenoma, or localization focal nodular hyperplasia). Patients diagnosed with intrahepatic cholangiocarcinoma, liver metastasis (from other tissues), or a combination of two or more kinds of tumors were excluded (Fig. S1A). For this study, primary liver cancer was defined during surgery, combined with preoperative contrast-enhanced CT according to the guidelines from the “Clinical Diagnosis and Staging Criteria for Primary Liver Cancer” [18]. The diagnosis of metastasis depended mostly upon abdominal enhanced CT and histopathological examination and includes lymph node metastasis, intra- and extra-hepatic metastases with/without bile duct or portal vein tumor thrombus. The patients were categorized according to the Barcelona Clinic Liver Cancer (BCLC) staging system (stage 0, A, B, C, and D), with Edmondson-Steiner (tumor differentiation) classification (I-II, III-IV) and Child–Pugh scoring (class A, B, and C) [19]. We collected tissue samples from 39 out of the 117 HCC patients, including 23 with metastasis and 16 without metastasis. These samples consisted of tumor, matched adjacent, and distal tissues. Furthermore, we had access to comprehensive clinical data for these patients, including information such as age, gender, serum HBsAg, HBV DNA levels, platelet count, albumin levels, ALT and AST enzyme levels, prothrombin time, AFP (alpha-fetoprotein) levels, PIVKA-II levels, and liver cirrhosis status.

### Establishment of HCC patient-derived xenograft (PDX) model

Human HCC tissues were obtained from the West China Hospital. The West China Hospital Institutional Review Committee (2019–905) approved the study protocol, and informed consent was obtained from the patients. The clinical details for the PDX donors are provided in Table S1. The HCC-PDX1 donor remained HCC-free for two years post-surgery, whereas the donor of PDX2 developed lung metastasis six months after surgery. HCC specimens were directly subcutaneously implanted in NOD/SCID mice (Nanjing University, CN); the PDX tumors were collected when they reached 300–500 mm^3^, evenly cut into small pieces, and re-implanted into the liver (left lobe) of NOD/SCID mice (anesthetized with a continuous supply of isoflurane and oxygen and cut with a median abdominal incision). The mice were sacrificed after 28 days, and endpoint anatomy was determined.

### Single-cell RNA-seq

#### Tissue dissociation and purification

HCC tissues from five patients (outside of the 117 HCC patients) with metastasis were processed immediately after resection to generate single-cell suspensions. Clinicopathological information for the five patients can be found in Table S2. Briefly, after harvested, tissues were washed in ice-cold RPMI1640 and dissociated using Demonstrated Protocol Adult Mouse Nuclei Isolation RevA (10 × Genomics Catalog No.CG000393 Rev A) from Miltenyi Biotec as instructions. DNase treatment was optional according to the viscosity of the homogenate. Cell count and viability was estimated using a fluorescence Cell Analyzer (Countstar Rigel S2) with AO/PI reagent after the removal of erythrocytes (Miltenyi 130–094-183), debris (Miltenyi 130–109-398) and dead cells (Miltenyi 130–090-101). Finally, fresh cells were washed twice in the RPMI1640 and then resuspended at 1 × 10^6^ cells per ml in 1 × PBS and 0.04% bovine serum albuminate.

#### Single-cell RNA-Seq library construction

Single-cell RNA-Seq libraries were prepared using Chromium Next GEM Single Cell 3′ Reagent Kits v3.1 (10 × Genomics). The indexed sequencing libraries were cleaned using SPRI beads, quantified by quantitative PCR (KAPA Biosystems KK4824), and sequenced on an Illumina NovaSeq 6000 (paired-end, 150bp reads).

#### Clustering and visualization

Seurat [20] was used to perform the clustering and visualization according to the following steps: 1) *Data normalization*. LogNormalize, a global-scaling normalization method, was employed to normalize the expression. The expression measurement of one transcript was divided by those of all the transcripts of the cell and multiplied by a scale factor (10,000 by default), and then the results were logarithmic transformed. 2) *Detection of highly variable features*. FindVariableFeatures was used to get 2,000 features per dataset. 3) *Scaling*. A linear transformation ('scaling'), a standard pre-processing step prior to dimensional reduction techniques, was applied. 4) *Dimensional reduction*. Principal component analysis (PCA) on the scaled data was performed, and the first 15 principal components were used in the following steps. 5) *Clustering*. A graph-based approach was applied to cluster the cells. 6) tSNE/UMAP. The non-linear dimensional reduction technique was used to visualize and explore these datasets. 7) *Cluster markers*. FindAllMarkers with the default parameters (except “logFC.threshold = 1”) was used to find markers that determined the cell clusters via differential expression, and the top 9 markers were visualized.

#### Cell trajectory analysis

The single-cell trajectory from LCSCs to matured HCC cells was reconstructed using Monocle2 (v2.12.0). Pseudo time was calculated using reduceDimension and orderCells, and trajectory patterns were determined through the differential Gene Test function. Highly variable genes along the trajectory were visualized using heatmaps that were generated with plot_pseudotime_heatmap.

#### InferCNV analysis and epithelial expression score

A scRNA-seq raw count matrix was used as input for the inferCNV package [21], with follicular cells derived from normal tissue as the normal reference. An epithelial expression score (the average from a 15-gene dataset) was used to identify malignant cells [22].

### Data analysis

HCC and normal tissue expression profiles were obtained from The Cancer Genome Atlas (TCGA) database, in which *NRCAM* expression was analyzed. The expression data was also cross-referenced with TCGA clinical data, and the Kaplan–Meier estimator was used to produce survival curves. Additionally, a Gene Set Enrichment Analysis (GSEA) was performed to produce enrichment scores (ES) that indicate the degree to which the specified gene sets are under/overrepresented in TCGA HCC patients. False discovery rates were calculated by comparing the data with 1000 Monte-Carlo simulations. The ES data was displayed using enrichment plots, which were compiled using the running ES for the specified gene set, the ranking within the list of all the analyzed protein-coding genes, and the value of the ranking metric as a measurement of the correlation of the gene with *NRCAM* expression (low/high).

### Establishment of murine oncogene-driven HCC model

#### Mice

C57BL/6 mice (male) were purchased from the Model Animal Research Center (Nanjing University, CN). All animal studies were performed in accordance with the guidelines and the approval of the Institutional Animal Care and Use Committee of Sichuan University. Orthotopic transplantation experiments with C57BL/6 organoids were transplanted into syngeneic C57BL/6 recipient mice.

#### Isolation of liver progenitor cells and culture of LPC organoids

Mice were fed a 0.1% 3,5-diethoxycarbonyl-1,4-dihydrocollidine (DDC) diet for four weeks to activate the liver progenitor cells (LPCs), which were isolated using cell sorting as previously described [23, 24]. Briefly, liver perfusion was performed using collagenase (Roche), the dissociated cells were suspended in DMEM, passed through a 100 μm nylon mesh, and centrifuged at 50 RCF (relative centrifugal force) for five minutes at 4°C. The supernatant was collected and centrifuged again at 300g for five minutes at 4°C. The LPCs were sorted using a MoFlo XDP machine after being stained with an anti-LGR5 antibody and an Alexa-594 conjugated goat anti-rabbit secondary antibody.

#### Retrovirus generation and shRNA cloning

The MIG-MYC-NRCAM plasmid was constructed in two steps using the MSCV-IRES-GFP (MIG) plasmid from Addgene (#20672). Firstly, MYC (isolated PCR fragment) was inserted into the plasmid using T4 DNA ligase, following digestion with EcoRI/BamHI. Secondly, the plasmid was digested with Not1 and Mlu1, into which a 3.8kb PCR NRCAM fragment was inserted using Gibson Assembly Master Mix (New England Biolabs; catalog number E2611S). The pcDNA6.2 plasmids containing shRNA sequences (in Table S3) were digested and ligated with T-intron, then ligated with MIG-MYC-Vec or MIG-MYC-NRCAM-Vec.

#### Retrovirus production

Retroviruses were produced via CaCl_2_ co-transfection of 293T cells with the relevant transfer vector and packaging plasmids (pcl-Eco). The supernatant was collected 36 and 48 h post-transfection and filtered with a 0.45μm filter, the viruses were concentrated by ultracentrifugation at 25,000 rpm for two hours and resuspended in culture medium.

#### Retroviral infection of LPC

The concentration of the LPCs was adjusted to 1 × 10^6^/mL, which were plated on 6-well plates with a 1:1 ratio of virus-containing medium (4mL total), with 1mg/mL polybrene (50 ×) and 1M HEPES (100 ×). The plates were centrifuged at 1000 RCF for 90 min at 37°C, the media was replaced after a three-hour incubation at 37°C with 5% CO_2_. The transduction efficiency was determined by assessing the ratio of GFP-expressing cells by flow cytometry (FCM).

#### Culture of murine MYC-driven LCSC organoids

Retroviral infected LPCs, including MIG-MYC, MIG-MYC-Vec (Vector), MIG-MYC-Con (Control), MIG-MYC-NRCAM (over-expression MYC and NRCAM), MIG-MYC-shNrcam (MYC overexpression, Nrcam knocked down), and MIG-MYC-NRCAM-shMacf1 (MYC and NRCAM overexpression, Macf1 knockdown) organoids were mixed with Matrigel (BD Bioscience, US) and cultured as previously described [23]. Briefly, the medium composition for the first three days was as follows: AdDMEM/F12 (Invitrogen, USA) supplemented with 0.5% B27 and 1% N2 (Gibico, US), N-acetylcysteine (1.25 mM, Sigma-Aldrich, US), gastrin (10nM, Sigma, US), EGF (50ng/mL, Peprotech, US), RSPO1 (50 ng/mL, R&D, US), FGF10 (100 ng/mL, Peprotech, USA), nicotinamide (10mM, Sigma-Aldrich, US), HGF (50ng/mL, Peprotech, US), 25ng/mL Noggin (R&D, USA), 25ng/mL WNT (R&D, US) and 10μM Y27632 (Sigma-Aldrich, US). Medium without Noggin, WNT, and Y27632 was used after three days (replaced every other day).

#### Construction of murine oncogene-driven HCC model in vivo

For the orthotopic organoid transplantations, mice were anesthetized with a continuous supply of isoflurane and oxygen and cut with a median abdominal incision, into which 1 × 10^5^ cells (50 μL single-cell suspension with disorganized organoids) were injected into the left lobe of the liver using a 27-gauge needle.

### Tissue clearing

#### Paraformaldehyde PFA fixation

Mice were anesthetized with a continuous supply of isoflurane and oxygen and perfused with ice-cold 1 × PBS followed by ice-cold 4% Paraformaldehyde solution. The dissected samples were incubated in 4% Paraformaldehyde at 4°C with gentle shaking overnight and washed twice at room temperature with 1 × PBS for two hours.

#### Tissue clearing, 3D imaging, and image processing

The fixed tissue samples were cleared using a Nuohai Tissue Clearing Kit (Cat#: NH210701, Nuohai Life ScienceCo., Ltd, CN) according to the manufacturer’s instructions. Imaging was performed using a Nuohai LS18 Tiling Light Sheet Microscope (Nuohai Life Science Co., Ltd, CN; laser lines: 405, 488, 561, 637 nm). A 1 × /0.25NA objective (Olympus MVPLAPO) was used to collect the fluorescence. The magnification of the microscope was set at 3.2 × , with a spatial resolution of approximately 4 × 4 × 10 μm^3^ [25]. The images were processed with LS18 ImageCombine software (Nuohai Life Science Co., Ltd, CN) and rendered using Amira (Thermo Fisher Scientific, US). Amira's Interactive Thresholding was used to isolate the tumors from the background tissue. Tumors with weak correlation or connection points were divided into different tumors. The number of tumors (and their volume) was calculated following Binary Smoothing.

### Multiplex immunofluorescent (mIF)

Multiplex immunofluorescent (mIF) was performed following the sequential binding of primary antibodies to 4-μm-thick formalin-fixed, paraffin-embedded whole tissue sections. A TSA 7-color kit (abs50015-100T, Absinbio) and DAPI (D1306; Thermo Fisher Scientific, US) were used according to the manufacturer's instructions. The slides were imaged using a Pannoramic MIDI Scanner (3DHISTECH) and analyzed using Indica Halo software (Indica Labs, US).

### Real-time quantitative PCR analysis

Total RNA was extracted from cells with TRIzol Reagent (Invitrogen, US). Total cellular RNA was reverse-transcribed using Moloney Murine Leukemia Virus (MMLV) reverse transcriptase (Gibco, US). Blank reactions with no RNA were performed in all experiments. The expression of genes mRNA was measured by real-time PCR using Maxima SYBR Green/ROX qPCR Master Mix (Thermo Fisher Scientific, US). The primer sequences for Actin/Gapdh (the reference) and candidate genes are listed in Table S4. The expression fold changes for the candidate genes relative to the reference gene were calculated using the normalized expression (ΔCt) method with default threshold values using CFX Manager Software (Bio-Rad, US).

### Western blotting analysis

The total protein content of cells and tissues was extracted with Radio Immunoprecipitation Assay (RIPA) lysis buffer (#9803, Cell Signaling Technology, US) following the manufacturer’s instructions; 50 μg of total protein was taken for electrophoresis and transferred onto polyvinylidene fluoride (PVDF) membranes (Millipore, US) using a wet transfer method. The PVDF membranes were blocked with 5% skimmed milk for 1.5 h, washed with TBST, incubated with primary antibodies, and shaken overnight at 4°C. The PVDF membranes were removed and washed with TBST and incubated with a secondary antibody at room temperature for an hour prior to being washed with TBST. The primary and secondary antibodies used for Western blotting are listed in Table S5. Immunodetection was performed using the ECL-Plus kit (Thermo Fisher Scientific, US) and analyzed with the Invitrogen iBright CL1500 Imaging System (Thermo Fisher Scientific, US). All of the blotting experiments were repeated in triplicate.

### Immunohistochemistry (IHC)

The immunohistochemical staining against NRCAM, ARG1, and Hep Par 1 was performed using formalin-fixed paraffin-embedded (FFPE) sections (which were de-paraffinized and rehydrated before staining). Following antigen retrieval, endogenous biotin activity was blocked with normal bovine serum, and the sections were incubated with the primary antibodies in a humidified chamber. Horseradish peroxidase-conjugated secondary antibody was applied to the sections, followed by incubation with DAB (3,3’-diamino-benzidine) substrate for color development (antibodies recorded in Table S5). The slides were subsequently dehydrated and mounted with Permount Mounting Medium (Fisher Scientific, US). The abundance of the molecules were estimated by microscopy (CX22, OLYMPUS, JP), and the images were captured using a digital pathology system Panoramic MIDI (3D Histech, HU) or a microimaging system (DM1000, Leica, DE). Immunohistochemical scores were calculated according to the Barnes scoring criteria, and differences between scores for the two groups were analyzed. Cell colony formation assay.

The cells were seeded (5 × 10^3^ per well) into a six-well tissue culture plate containing DMEM medium; the cells were irradiated after six hours. The colony formation potential for the cells was determined by crystal violet staining following a 10 to 14-day incubation. Clones containing more than 50 cells were scored as survivors [26].

### Wound healing assay

Mouse LCSCs were inoculated in 6-well plates with a density of 5 × 10^5^ cells/mL for transfection (in triplicate). One or two scratches in the middle of the bottom of each well with a sterile pipette tip, the cells were subsequently washed with PBS three times, and 2mL DMEM was added. The cells were imaged using an inverted at 0, 24, and 48 h after scratching. The images were analyzed by Image J (National Institutes of Health, US) to compare the number of cells crossing the scratch versus the control group, the percentage of wound closure was calculated to reflect the level of cell migration.

### Transwell assay for the detection of cell invasion

Matrigel was dissolved overnight at 4°C and diluted with serum-free medium (SFM) (Thermo Fisher Scientific, US) at a ratio of 1:7. Matrigel invasion chambers were placed in the wells of a sterile 24-well plate, and 20 μL Matrigel was added into the chamber (ensuring that it evenly covered the chamber bottom). Following transfection (24 h), single-cell suspensions (1 × 10^5^ cells/mL) were produced in serum-free DMEM. The cells were added to the upper chamber of each well for transwell assay, with the 600 μL medium containing 10% fetal bovine serum (Thermo Fisher Scientific, US) in the lower chamber and maintained for 24 h at 37°C in a humidified incubator with 5% CO_2_. The media was removed from the upper chamber, and cells that did not penetrate the membrane were removed using a wet cotton swab. The chamber was washed three times with PBS and fixed with 4% paraformaldehyde for 30 min. Crystal violet staining was performed (20-min incubation), and the chambers were imaged using an inverted microscope. Five visual fields were randomly selected, and the number of cells penetrating the stromal membrane were counted.

### Statistical analysis

All statistical analyses were done with the SPSS Version 18.0 (SPSS, US). The figures were drawn using GraphPad Prism 6 (GraphPad Software Inc., USA). *P* < 0.05 was used as the threshold for determining the significance of differences between groups. Quantitative variables were expressed using the mean plus/minus ( ±) the standard deviation or the median with interquartile ranges, while categorical variables were expressed with absolute and relative frequencies. T-tests or Wilcoxon tests were performed to determine the significance of differences between quantitative datasets, whereas the Chi-Square (χ2) test was used for qualitative data. Where relevant, the correlation between characteristics was calculated using Kendall’s Tau or Spearman’s Rank Correlation Coefficient. The P value was indicated in the figures using the following standard abbreviation: *P* > 0.05 (NS), *P* < 0.05 (*), *P* < 0.01 (**), *P* < 0.001 (***), *P* < 0.0001 (****).

Further details regarding the materials and methods can be found in the supplementary information.

## Results

### NRCAM is closely related to hepatocarcinogenesis and HCC metastasis

**Fig. 1:**
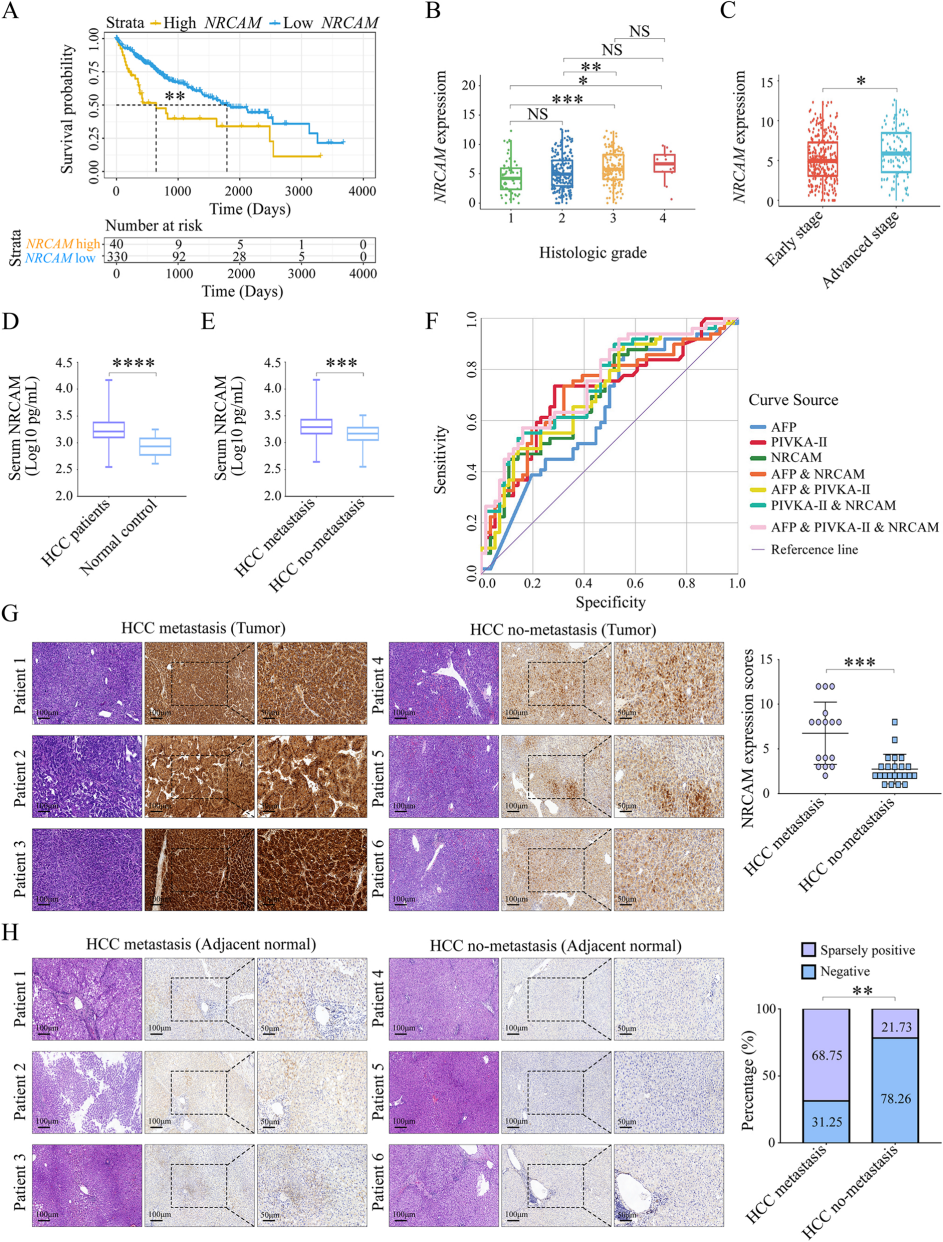
NRCAM expression is related to hepatocarcinogenesis and HCC metastasis. **A** Kaplan–Meier estimates of disease-free survival according to *NRCAM* status (high and low) for the cancer genome atlas (TCGA) database samples. **B** *NRCAM* expression for different histologic grades. **C** *NRCAM* expression at different HCC stages. **D** Serum NRCAM levels in HCC patients and non-HCC patients detected by ELISA. **E** Serum NRCAM levels in metastatic and non-metastatic HCC patients were detected using ELISA. **F** ROC curves evaluating the predictive value (for HCC metastasis) of serum AFP, PIVKA-II, and NRCAM levels. **G** IHC staining for NRCAM in HCC tumor tissue. **H** IHC staining for NRCAM in adjacent normal tissue. Data are represented as mean ± SD. *P* > 0.05 (NS), *P* < 0.05 (*), *P* < 0.01 (**), *P* < 0.001 (***)

*NRCAM* expression was higher in HCC than in normal tissue from TCGA patients (Table S6). The disease-free status was assessed following the stratification based upon *NRCAM* status (high/low expression). The survival time for patients with high *NRCAM* expression was significantly shorter than for patients with low expression (*P* = 0.001) (Fig. 1A). Additionally, *NRCAM* expression was significantly increased in tissues associated with a higher histologic grade (Fig. 1B) and in patients with advanced HCC (Fig. 1C). This data suggests that high *NRCAM* expression is associated with a poorer prognosis for HCC patients.

To explore the relationship between NRCAM and HCC, a total of 146 (117 HCC and 29 non-HCC) patients with focal liver lesions were collected, barcoded, and analyzed (see Additional file 1 for more details). There was no statistical difference (*P* > 0.05) between the age, gender, and albumin of the HCC and non-HCC patients. However, HCC patients had a higher rate of HCC-associated etiologies, including HBV infection, HCV infection, non-alcoholic fatty liver disease, alcoholic liver disease, autoimmune hepatitis (76.92% vs. 27.59%, *P* = 0.002), a higher rate of cirrhosis (59.83% vs. 17.24%, *P* < 0.001), a higher rate of platelet depletion (34.19% vs. 13.79%, *P* = 0.041), increased serum AFP (1307.22 ± 7229.01 vs. 7.18 ± 19.89 ng/ml, *P* = 0.048), and protein induced by Vitamin K absence/antagonist-II (PIVKA-II) levels (7031.90 ± 17,062.84 vs. 176.75 ± 812.60 mAU/ml, *P* < 0.001) (Table 1). In addition, the serum NRCAM levels in HCC patients were significantly higher than the non-HCC patients (3.23 ± 0.24 vs. 2.93 ± 0.19 Log10 pg/ml, *P* < 0.001) (Fig. 1D). The same was true for HCC patients with metastasis (3.31 ± 0.25 vs. 3.15 ± 0.20 Log10 pg/ml, *P* < 0.001) when compared to those without metastasis (Fig. 1E, Table 2). These preliminary results indicate that serum NRCAM levels significantly increased in patients with HCC and further increased in those with metastasis.

**Table 1:** Baseline characteristics of included participants

Characteristics	HCC (*N* = 117)	non-HCC (*N* = 29)	*P* value	Sig
Age (years)	54.25 ± 11.45	52.69 ± 11.46	0.516	
Gender				
Male	91	17	0.056	
Female	26	12		
Etiology				
Unknown etiology	27	21	0.002	**
HBV	75	6		
HCV	4	0		
NAFLD	4	2		
ALD	5	0		
AIH	2	0		
Liver cirrhosis				
Yes	70	5	< 0.001	***
No	47	24		
ALT (U/L)				
≥ 48	28	2	0.043	*
< 48	89	27		
AST (U/L)				
≥ 50	34	2	0.015	*
< 50	83	27		
Platelets (10^3^/mm^3^)				
≥ 100	77	25	0.041	*
< 100	40	4		
Albumin (g/L)	41.97 ± 5.51	41.56 ± 4.16	0.707	
AFP (ng/ml)	1307.22 ± 7229.01	7.139 ± 19.90	0.048	*
PIVKA-II (mAU/ml)	7031.90 ± 17,062.84	176.76 ± 812.60	< 0.001	***
NRCAM (pg/ml)	2024.11 ± 1818.41	920.10 ± 398.76	< 0.001	***

Significance (Sig): *P* < 0.05 (*), *P* < 0.01 (**), *P* < 0.001 (***)

**Table 2:** Serum NRCAM levels in 117 HCC patients

Clinic Characteristics	N	Serum NRCAM level (pg/ml)	*P* value	Sig
AGE (years)				
≤ 55	60	1971.56 ± 1889.16	0.762	
> 55	57	2074.02 ± 1763.09		
Gender				
Male	91	2014.50 ± 1895.07	0.915	
Female	26	2057.73 ± 1553.24		
Etiology				
Unknown etiology	27	1789.04 ± 964.12	0.872	
HBV	75	2140.97 ± 2173.04		
HCV	4	2293.70 ± 528.13		
NAFLD	4	2099.82 ± 627.06		
ALD	5	1206.95 ± 516.13		
AIH	2	2167.12 ± 819.50		
BCLC stages				
0-A	71	1795.41 ± 1474.33	0.091	
B-C	46	2377.09 ± 2220.94		
Number of nodules				
Single	62	1809.14 ± 1576.42	0.173	
Multiple	53	2277.10 ± 2078.26		
Tumor differentiation				
Well/moderately	68	1958.56 ± 1919.68	0.648	
Poorly	49	2115.07 ± 1683.02		
Metastasis				
Yes	56	2527.92 ± 2448.06	0.004	**
No	61	1561.59 ± 666.95		

Significance (Sig): *P* < 0.01 (**)

Receiver Operating Characteristic (ROC) curves were calculated for serum AFP, PIVKA-II, and NRCAM to evaluate diagnostic and predictive value for HCC and metastasis, respectively. The serum NRCAM levels (area under the curve –AUC 0.84, 95% CI 0.77–0.92, *P* < 0.001) displayed moderate diagnostic value for HCC, which was similar to AFP (AUC 0.86, 95% CI 0.79–0.92, *P* < 0.001) and PIVKA-II (AUC 0.86, 95% CI 0.79–0.94, *P* < 0.001). Moreover, the three biomarkers increased the AUC to 0.91 (95% CI 0.86–0.96, *P* < 0.001) when combined (Fig. S1B, Table S7). Serum NRCAM (AUC 0.70, 95% CI 0.60–0.80, *P* < 0.001), akin to AFP (AUC 0.63, 95% CI 0.52–0.74, *P* = 0.021) and PIVKA-II (AUC 0.71, 95% CI 0.60–0.81, *P* < 0.001), also displayed moderate predictive accuracy for HCC metastasis. The AUC was increased to 0.75 (95% CI 0.66–0.85, *P* < 0.001) when NRCAM, AFP, and PIVKA-II were combined (Fig. 1F, Table 3). Ultimately, the elevation of NRCAM levels in serum provided an independent predictive value for HCC diagnosis and metastasis (Tables 4 and 5, S8).

**Table 3:** ROC curves for AFP, PIVKA-II, and NRCAM when predicting HCC metastasis

Factors	AUC (95% CI)	*P* value	Sig
AFP	0.63 (0.52–0.74)	0.021	*
PIVKA-II	0.71 (0.60–0.81)	< 0.001	***
NRCAM	0.70 (0.60–0.80)	< 0.001	***
AFP & NRCAM	0.71 (0.61–0.81)	< 0.001	***
AFP & PIVKA-II	0.71 (0.61–0.82)	< 0.001	***
PIVKAII & NRCAM	0.74 (0.65–0.84)	< 0.001	***
AFP & PIVKAII & NRCAM	0.75 (0.66–0.85)	< 0.001	***

Significance (Sig): *P* < 0.05 (*), *P* < 0.001 (***)

**Table 4:** Univariate and multivariate logistic analysis in HCC patients with metastasis

Clinic Characteristics	Univariate		multivariate		
	**Odds ratio (95% CI)**	***P* value**	**Odds ratio (95% CI)**	***P* value**	**Sig**
Age (years)	0.96 (0.93–0.99)	0.021	0.97 (0.92–1.03)	0.301	
Gender	1.09 (0.46–2.62)	0.843			
Etiology					
Unknown etiology	Reference				
HBV	1.22 (0.50–2.95)	0.663			
HCV	3.75 (0.35–40.81)	0.278			
NAFLD	1.25 (0.15–10.23)	0.835			
ALD	0.31 (0.03–3.18)	0.326			
AIH	1.25 (0.07–22.13)	0.879			
Detectable HBV DNA viral load	3.25 (1.49–7.05)	0.003	3.95 (1.13–13.78)	0.031	*
Liver cirrhosis	1.43 (0.68–3.01)	0.347			
Tumor size (cm)	1.17 (1.05–1.30)	0.005	1.00 (0.81–1.22)	0.974	
Number of nodules					
Single	Reference				
Multiple	8.92 (3.88–20.50)	< 0.001	34.92 (7.66–159.10)	< 0.001	***
Tumor differentiation					
Well/moderately	Reference				
Poorly	4.04 (1.85–8.81)	< 0.001	3.14 (0.97–10.17)	0.056	
Vascular invasion	5.41 (2.01–14.55)	0.001	24.26 (3.96–148.46)	0.001	**
BCLC stages					
0-A	Reference				
B-C	11.25 (4.58–27.62)	< 0.001	3.42 (0.74–15.89)	0.116	
Platelets (10^3^/mm^3^)	1.01 (1.00–1.01)	0.038	1.01 (1.00–1.02)	0.022	*
ALT (U/L)	1.00 (0.99–1.01)	0.614			
AST (U/L)	1.01 (1.00–1.02)	0.238			
Albumin (g/L)	0.91 (0.84–0.98)	0.014	0.98 (0.89–1.08)	0.643	
Birrirubin (umol/L)	1.02 (0.97–1.07)	0.407			
PT (s)	1.17 (0.83–1.66)	0.378			
AFP (ng/ml)	1.00 (1.00–1.00)	0.458			
PIVKA-II (mAU/ml)	1.00 (1.00–1.00)	0.014	1.00 (1.00–1.00)	0.779	
NRCAM (pg/ml)	1.00 (1.00–1.00)	0.002	1.00 (1.00–1.00)	0.007	**

Significance (Sig): *P* < 0.05 (*), *P* < 0.01 (**), *P* < 0.001 (***)

*NAFLD* non-alcoholic fatty liver disease, *ALD* Alcoholic liver disease, *AIH* Autoimmune Hepatitis

**Table 5:** ROC curves for HCC diagnosis and metastasis

Characteristics	ROC	ROC *P* value	Cut-off	Se	Sp
Diagnosis HCC					
AFP	0.86 (0.79–0.92)	< 0.001	8.01	74.11%	97.13%
PIVKA-II	0.86 (0.79–0.94)	< 0.001	92.14	66.88%	94.58%
NRCAM	0.84 (0.77–0.92)	< 0.001	1091.93	88.38%	76.16%
Metastasis prediction					
AFP	0.63 (0.52–0.74)	0.021	7.23	91.22%	47.46%
PIVKA-II	0.71 (0.60–0.81)	< 0.001	229.43	74.18%	78.53%
NRCAM	0.70 (0.60–0.80)	< 0.001	2074.21	49.39%	89.88%

*Se* sensitivity, *Sp* specificity, *ROC* receiver operating curve

To further investigate the relationship between NRCAM production and HCC metastasis, 39 HCC tissue samples (16 and 23 HCC cases with and without metastasis, respectively) (Fig. S1C, D) were stained with an anti-NRCAM antibody. NRCAM staining was higher in metastatic samples (those with a high Barnes score) than in the non-metastatic samples (6.75 ± 3.49 vs. 2.74 ± 1.66, *P* < 0.001) (Fig. 1G). Moreover, trace NRCAM expression was detected in the adjacent normal tissue for many metastatic HCC cases, especially in the portal area. In contrast, adjacent normal tissues from HCC cases without metastasis were generally not NRCAM positive (*P* = 0.007) (Fig. 1H). These results support the notion that cells with high NRCAM expression were more likely to be associated with malignant tissues and greater migrative potential.

### NRCAM influences metastatic traits in murine HCC-PDX models

**Fig. 2:**
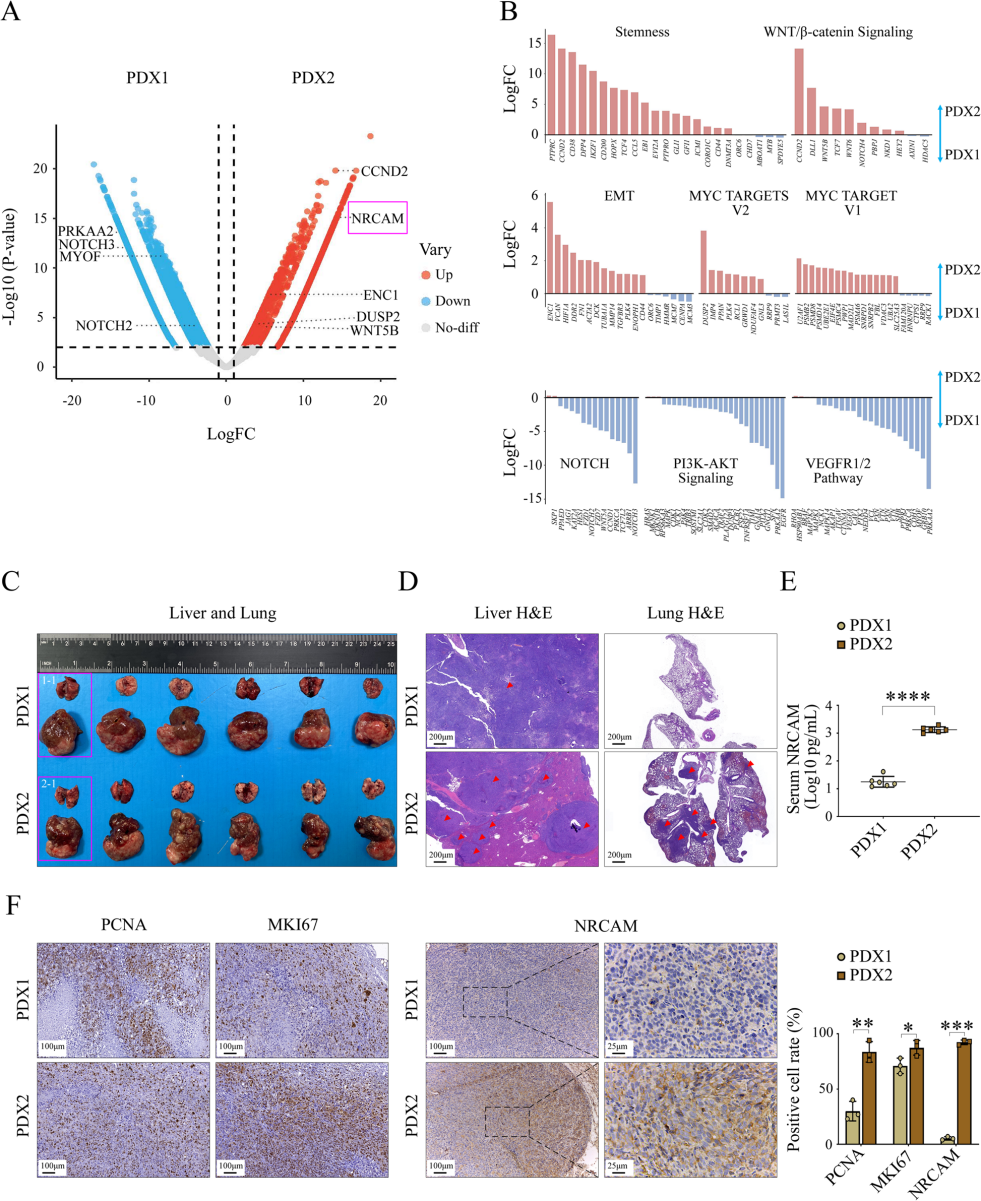
HCC-PDX models with different NRCAM expression levels. **A** Normalized volcano plot showing the differentially expressed genes (fold change > 1 and *P* =  < 0.05) between PDX1 and PDX2 tumor tissue (downregulated genes in blue; upregulated genes in red; not significantly different genes in grey). **B** Genes involved in Stemness, WNT/β-catenin signaling pathway, EMT, MYC targets V1, MYC targets V2, NOTCH signaling pathway, PI3K-AKT signaling pathway, and VEGFR1/2 signaling pathway expression levels in PDX1 and PDX2 tumor. Genes highly expressed in PDX1 are blue, and those highly expressed in PDX2 are red. **C** Mouse lungs and livers 28 days after the tumor was implanted in the PDX1 and PDX2 groups. **D** Liver and lung H&E staining for PDX1 (Mouse: 1–1) and PDX2 (Mouse: 2–1) groups. Red arrows indicate tumor tissue in the livers and lungs. **E** Serum NRCAM levels in PDX1 and PDX2 groups were detected using ELISA. Blood was tested for six mice from each group. Data are represented as mean ± SD. **F** IHC staining for PCNA, MKI67, and NRCAM in PDX1 and PDX2 tumor tissue. Data are represented as mean ± SD. *P* < 0.05 (*), *P* < 0.01 (**), *P* < 0.001 (***), ****, *P* < 0.0001

We further explored the relationship of NRCAM expression in HCC metastasis using two HBV-PDX models in vivo. RNA sequencing (RNAseq) of PDX tumor (first generation) confirmed that PDX2 tumor tissue had a higher *NRCAM* expression level compared to PDX1 tumor tissue (Fig. 2A). In addition, genes involved in Stemness, WNT/β-catenin signaling pathway, EMT, and MYC targets V1&2 had increased expression in PDX2, whereas genes in the NOTCH signaling pathway, PI3K-AKT signaling pathway, and VEGFR1/2 signaling pathway were enriched in PDX1 (Fig. 2B).

Substantial differences were observed between the mice implanted with PDX1 and PDX2, with the latter being associated with significantly more liver tumors (Fig. 2C). Moreover, lung metastasis was observed in the mice with PDX2 tissue but not the PDX1 group (Fig. 2D). The serum NRCAM levels in PDX2 group were significantly higher than the PDX1 group (3.12 ± 0.10 vs. 1.25 ± 0.19 Log10 pg/ml, *P* < 0.0001) (Fig. 2E). Livers from PDX1 and PDX2 groups were stained with anti-PNCA, anti-MKI67 (Ki-67), and anti-NRCAM antibody. The rate of positive staining for PNCA (83.67 ± 9.29 vs. 30.00 ± 8.89, *P* = 0.002), MKI67 (87.33 ± 6.66 vs. 71.00 ± 7.00, *P* = 0.043), and NRCAM (92.33 ± 2.08 vs. 5.33 ± 1.53, *P* < 0.0001) was higher in PDX2 cells. These findings reinforce the idea that high NRCAM expression in HCC is indicative of metastatic characteristics.

### *NRCAM* expression was correlated with *MYC* in LCSCs

Tumor tissue from five patients with HCC metastasis was profiled using scRNA-seq to explore the biological characteristics associated with elevated *NRCAM* expression (Table S1, Fig. S2A). Transcriptomes from 36,085 single cells were used for a differential gene expression analysis to identify cluster-specific markers. In total, 23 main clusters were identified (Fig. S2B). Well-established canonical markers distinguished different cell clusters, such as hepatocytes (*KRT18*, *MASP2*, *HPR*, and *ALB*), hepatic stellate cells (*ACTA2*, *PDGFRB*, *RGS5*, and *COL1A1*), T-cells (*CD3E*, *CD3G*, and *PTPCR*), macrophages (*C1QA* and *C1QB*), neutrophils (*FCGR3B* and *CXCL8*) (Fig. S2C). In total, 9663 hepatocytes were identified, which were divided into eight distinct clusters (2, 3, 6, 8, 16, 17, 19, and 20) (Fig. S2D) representing hepatocyte heterogeneity. A cell trajectory analysis was performed (Fig. S2E) to enable LCSC identification, i.e., those expressing *EPCAM* and *KRT18*, which were localized at the start (stages 1 and 2) of the trajectory (Fig. S3A). Mature HCCs were represented in the latter trajectory stages (stages 3, 4, 5, 6, 7) and expressed adult liver markers [27], including *TAT*, *TDO2*, *SSTR2*, *CYP7A1*, *CYP3A4* and *CYP2B6* (Fig. S3B).

**Fig. 3:**
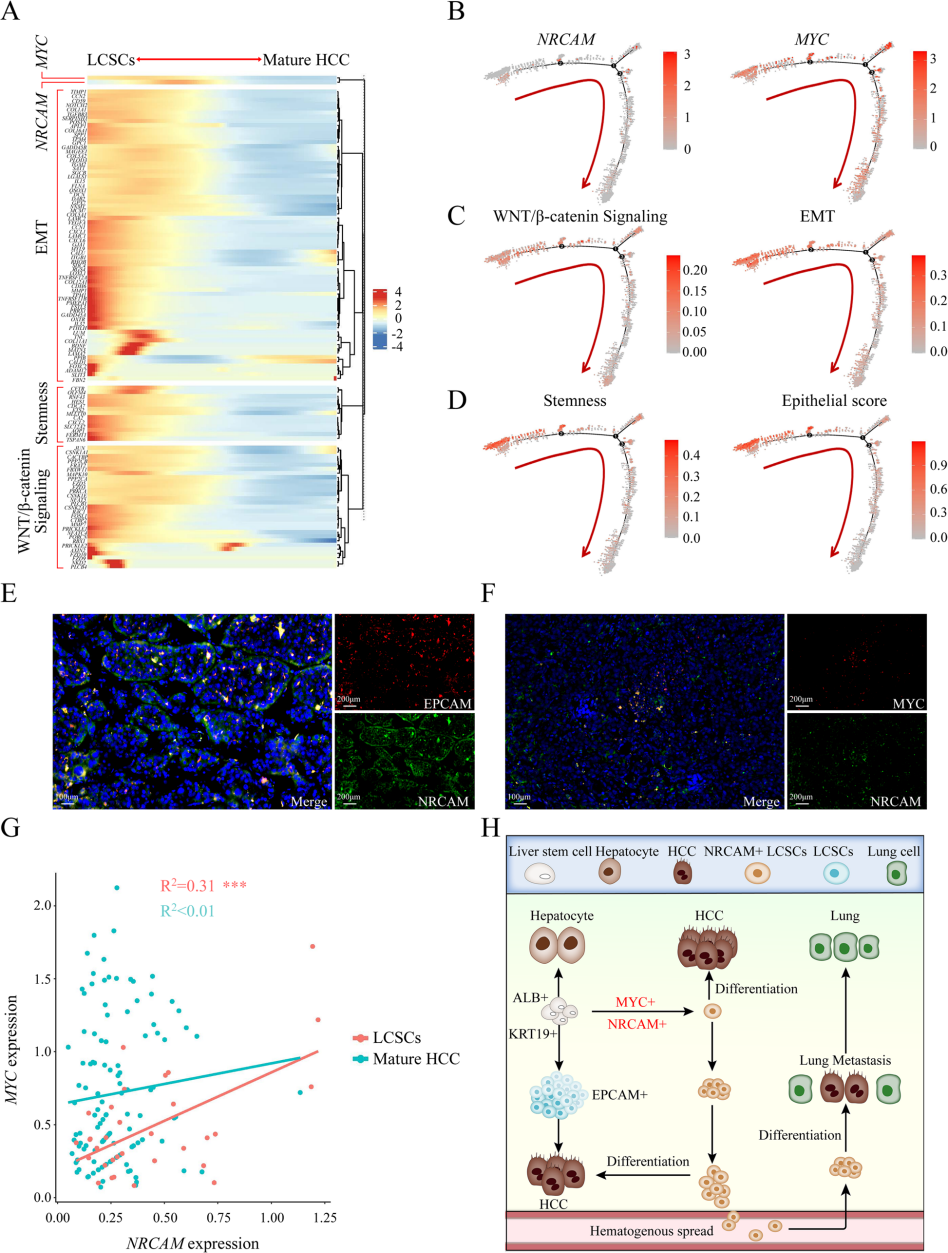
NRCAM was highly expressed and correlated with MYC in LCSCs. **A** A heatmap showing expression (color scale indicating Z-score) changes for 114 highly variable genes identified along the LCSCs-to-mature HCC trajectory. Significantly enriched functional annotations are also presented. **B** *NRCAM* and *MYC* expression across the trajectory. **C** Average WNT/β-catenin Signaling (151 genes) and EMT (200 genes) activation across the trajectory. **D** Average stemness (stem score, 35 genes) and epithelial gene (15 genes) expression score across the trajectory. Trajectory color scales represent relative expression. The arrows demonstrate potential cell-level evolutionary trajectory for B, C, and D. **E** IF staining to identify EPCAM (red) and NRCAM (green) double-stained cells in metastasic HCC tissue. **F** IF staining to identify MYC (red) and NRCAM (green) double-stained cells in metastatic HCC tissue. **G** *NRCAM* and *MYC* expression correlation. *NRCAM* expression was correlated with *MYC* in LCSCs (R = 0.31, P < 0.001) but not mature HCCs. **H** Diagram demonstrating the relevance of high *NRCAM* expression and *MYC* activation for LCSCs and HCC metastasis. *P* < 0.001 (***)

Expression changes for 114 highly variable genes were detected along the maturation axis from LCSCs to matured HCC, which included *NRCAM*, *MYC*, 70 genes associated with epithelial-mesenchymal transition, 13 genes related to stemness and 29 genes in the WNT/β-catenin signaling pathway (Fig. 3A). *NRCAM* was highly expressed in LCSCs with *MYC* activation (Fig. 3B). LCSCs also had an enrichment of genes with higher expression that were associated with EMT and the WNT/β-catenin signaling pathway (Fig. 3C). The stemness and epithelial scores for LCSCs were higher (Fig. 3D), which implied that LCSCs had features associated with a greater degree of malignancy compared to mature HCCs (Fig. S3C-F).

Immunofluorescence demonstrated that NRCAM and EPCAM were co-located in HCC cells (Fig. 3E); NRCAM and MYC were co-located in some HCC cells (Fig. 3F). Indeed, *NRCAM* expression was correlated with *MYC* in LCSCs, (R = 0.31, *P* < 0.001) but not mature HCCs (Fig. 3G). A similar result was observed using TCGA data, where higher *NRCAM* expression was associated with higher expression of stemness-associated genes (Fig. S3G). Furthermore, *NRCAM* expression was notably elevated in TCGA tissues where *MYC* was activated (Fig. S3H). A significant association (*P* = 0.01) was found between the expression of these two genes, evidenced by a correlation coefficient of R = 0.13 (Fig. S3I). These findings support the positive correlation between *NRCAM* and *MYC* expression in LCSCs (Fig. 3H).

### NRCAM promoted LCSC invasion and migration in organoids and allograft tumors

**Fig. 4:**
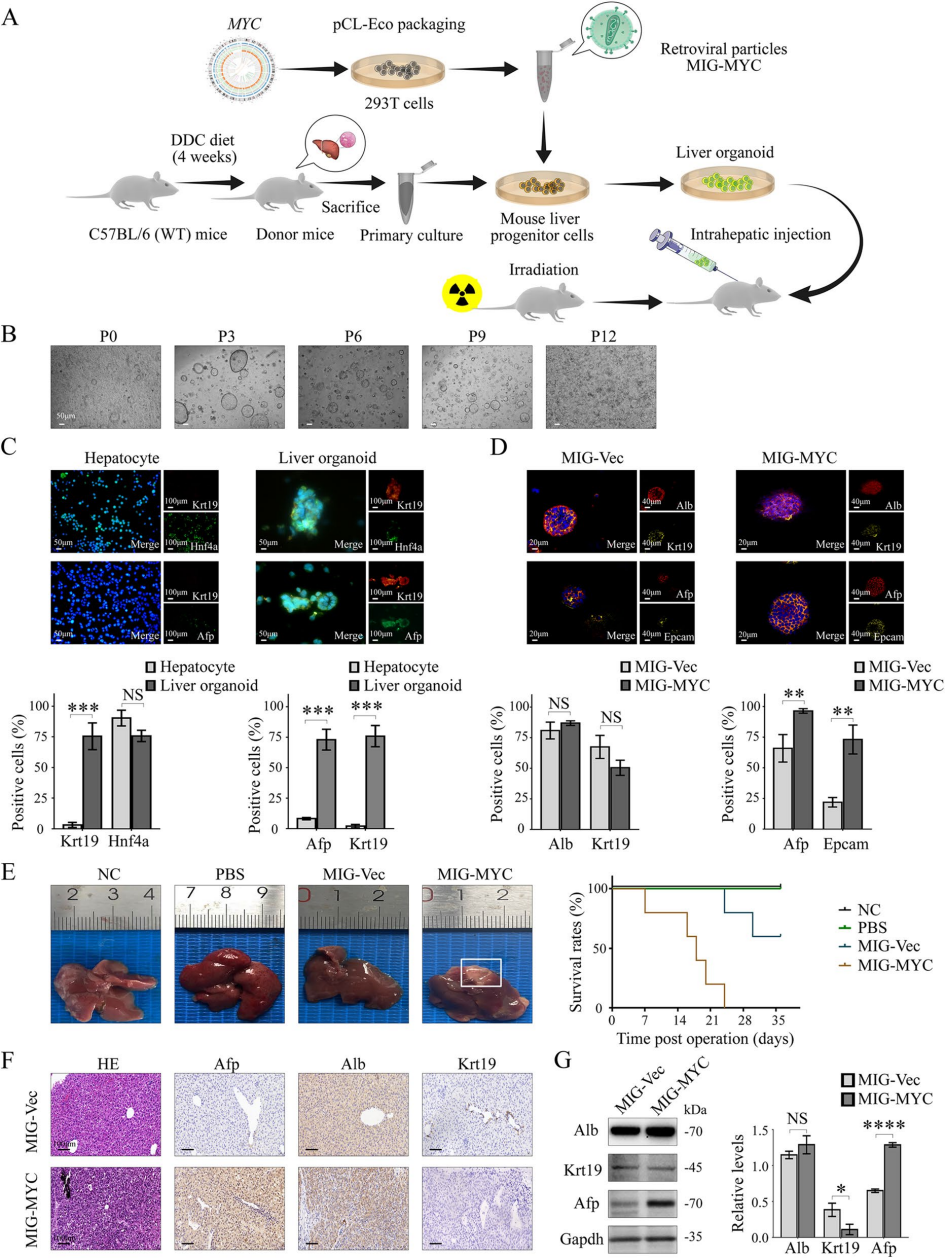
Murine oncogene-driven HCC allograft model. **A** schematic describing the creation of LCSCs organoid cultures from C57BL/6 (WT) mice liver stem cells and the establishment of an oncogene-driven murine liver cancer model. DDC: 0.1% 3,5-diethoxycarbonyl-1,4-dihydrocollidine. **B** Liver organoids are maintained and propagated in 3D culture. **C** IF staining and positive cell rates for Krt19, Hnf4a, and Afp in liver organoid and hepatocyte. **D** IF staining and positive cell rates for Alb, Krt19, Afp, and Epcam in MIG-Vec and MIG-MYC organoids. **E** MIG-MYC organoids formed obvious tumors in the C57BL/6 mice livers (the survival curves for MIG-vec and MIG-MYC organoid-driven allograft models). **F** IHC for Afp, Alb, and Krt19 in mice liver tissues. **G** Western blotting for Afp, Alb, and Krt19 in mice liver tissues. Data are represented as mean ± SD. *P* > 0.05 (NS), *P* =  < 0.05 (*), *P* =  < 0.01 (**), *P* =  < 0.001 (***), *P* =  < 0.0001 (****)

To determine whether NRCAM was the key factor affecting HCC metastasis, three-dimensional (3D) LCSC organoid cultures were established to produce a murine oncogene-driven HCC model (Fig. 4A). C57BL/6 donor mice were fed a 0.1% 3,5-diethoxycarbonyl-1,4-dihydrocollidine (DDC) diet for four weeks to promote progenitor cell proliferation. The progenitor cells were harvested from donor mice liver and were maintained and propagated in 3D culture (Fig. 4B). The organoids displayed a bi-potent liver stem cell phenotype, with Krt19, significantly increased Afp expression (compared to hepatocytes) and slightly decreased Hnf4a expression (Fig. 4C).

Retrovirus containing the oncogene *MYC* (Fig. 4D) were used to transform liver progenitor cells into LCSCs, which formed MIG-MYC organoids for 3D culture. While Alb levels remained unchanged, a slight decrease was observed in Krt19 in MIG-MYC organoids compared to MIG-Vec organoids. In contrast, the expression of Afp and Epcam significantly increased (Fig. 4D). The MIG-MYC organoids demonstrated an HCC-like phenotype and were transplanted into irradiation-induced transient immune deficient C57BL/6 mice (via intrahepatic injection). Upon examination 28 days post-allograft, these organoids were classified as malignant due to the formation of palpable liver tumors and their association with reduced survival (*P* < 0.0001) (Fig. 4E). IHC (Fig. 4F) and western blotting (Fig. 4G) revealed that the allografted tumors had high levels of Afp and Alb, but rarely displayed Krt19. These results verified that the MIG-MYC organoids differentiated into HCC in C57BL/6 (WT) mice.

**Fig. 5:**
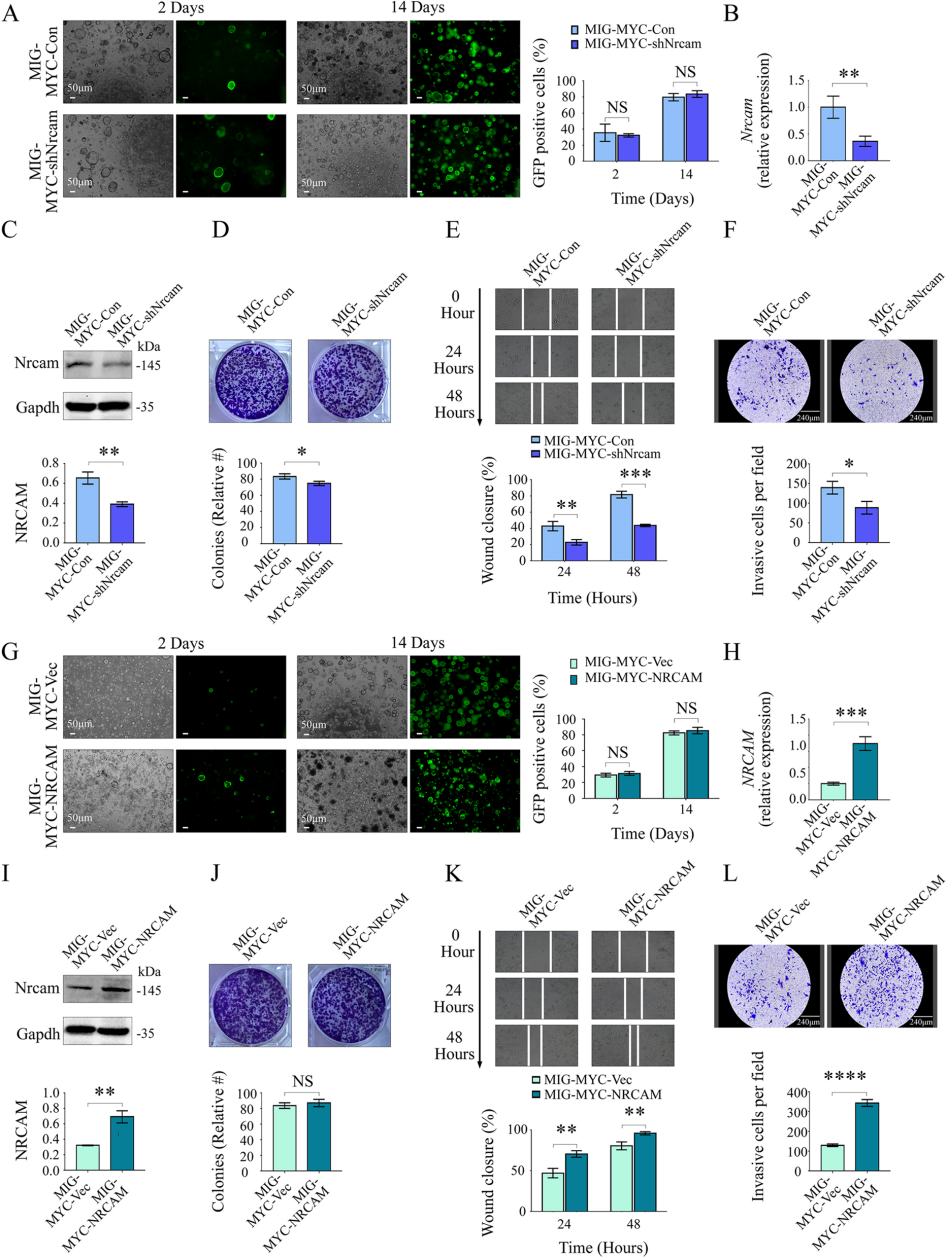
In vitro effects of NRCAM expression on LCSCs organoid migration and invasion. **A** GFP fluorescence for MIG-MYC-Con/MIG-MYC-shNrcam organoids two/14 days following transfection. **B** *Nrcam* expression in MIG-MYC-Con/MIG-MYC-shNrcam organoids. **C** Nrcam western blotting for MIG-MYC-Con/MIG-MYC-shNrcam organoids. **D** colony formation, **E** scratch, and **F** transwell assays for MIG-MYC-Con/MIG-MYC-shNrcam organoids. **G** GFP fluorescence of MIG-MYC-Vec and MIG-MYC-NRCAM organoids two and 14 days following transfection. **H** Expression of *NRCAM* in MIG-MYC-Vec and MIG-MYC-NRCAM organoids. **I** NRCAM western blotting for MIG-MYC-Vec and MIG-MYC-NRCAM organoids. **J** colony formation, **K** scratch, and **L** transwell assays for MIG-MYC-Vec and MIG-MYC-NRCAM organoids. Data are represented as mean ± SD (*n* = 3). *P* > 0.05 (NS), *P* < 0.05 (*), *P* < 0.01 (**), *P* < 0.001 (***), *P* < 0.0001 (****)

Next, Nrcam was knocked down, and NRCAM was overexpressed in MIG-MYC organoids (Fig. S4). There was no significant difference in transfection efficiency between MIG-MYC-Con and MIG-MYC-shNrcam organoids two and 14 days post-transduction (Fig. 5A). The MIG-MYC-shNrcam organoids displayed lower Nrcam levels than the MIG-MYC-Con organoids (Fig. 5B, C). A clone formation assay revealed that the MIG-MYC-shNrcam organoids formed slightly fewer colonies when compared to the MIG-MYC-Con organoids (83.67 ± 3.20 vs. 75.00 ± 2.65, P = 0.023) (Fig. 5D). Wound healing assays (Fig. 5E) also showed that the mean cell migration rate for the MIG-MYC-shNrcam (versus MIG-MYC-Con) organoids was markedly decreased at 24 h (0.43 ± 0.06 vs. 0.23 ± 0.03, *P* = 0.007) and 48 h (0.82 ± 0.04 vs. 0.44 ± 0.02, *P* < 0.001). Furthermore, a transwell assay (Fig. 5F) indicated that MIG-MYC-shNrcam organoid cells migrated at a significantly lower rate than MIG-MYC-Con organoids (140 ± 16/field vs. 89 ± 16/field, *P* = 0.018).

Like the MIG-MYC-shNrcam organoids (versus MIG-MYC-Con), the MIG-MYC-NRCAM organoids demonstrated no significant difference in transfection efficiency when compared to MIG-MYC-Vec two and 14 days post-transduction (Fig. 5G). qPCR and Western blotting confirmed that NRCAM levels were effectively increased in MIG-MYC-NRCAM organoids (Fig. 5H, I). A plate-based clone formation assay also showed that the MIG-MYC-Vec and MIG-MYC-NRCAM organoids (Fig. 5J) did not produce significantly different relative colony numbers (84.00 ± 3.61 vs. 87.33 ± 4.73, *P* = 0.386). However, the scratch assay showed that the mean MIG-MYC-NRCAM organoid migration rate was markedly increased at 24 h (0.47 ± 0.06 vs. 0.71 ± 0.04, *P* = 0.005) and 48 h (0.81 ± 0.05vs. 0.96 ± 0.02, *P* = 0.007) compared to the MIG-MYC-Vec organoids (Fig. 5K). A transwell assay also demonstrated the increased migrative capacity of the MIG-MYC-NRCAM organoids (Fig. 5L), which had higher transmembrane potential compared to MIG-MYC-Vec organoids (131 ± 7/field vs. 344 ± 17/field, *P* < 0.0001). These results suggested that NRCAM significantly affected LCSC invasion and migration.

**Fig. 6:**
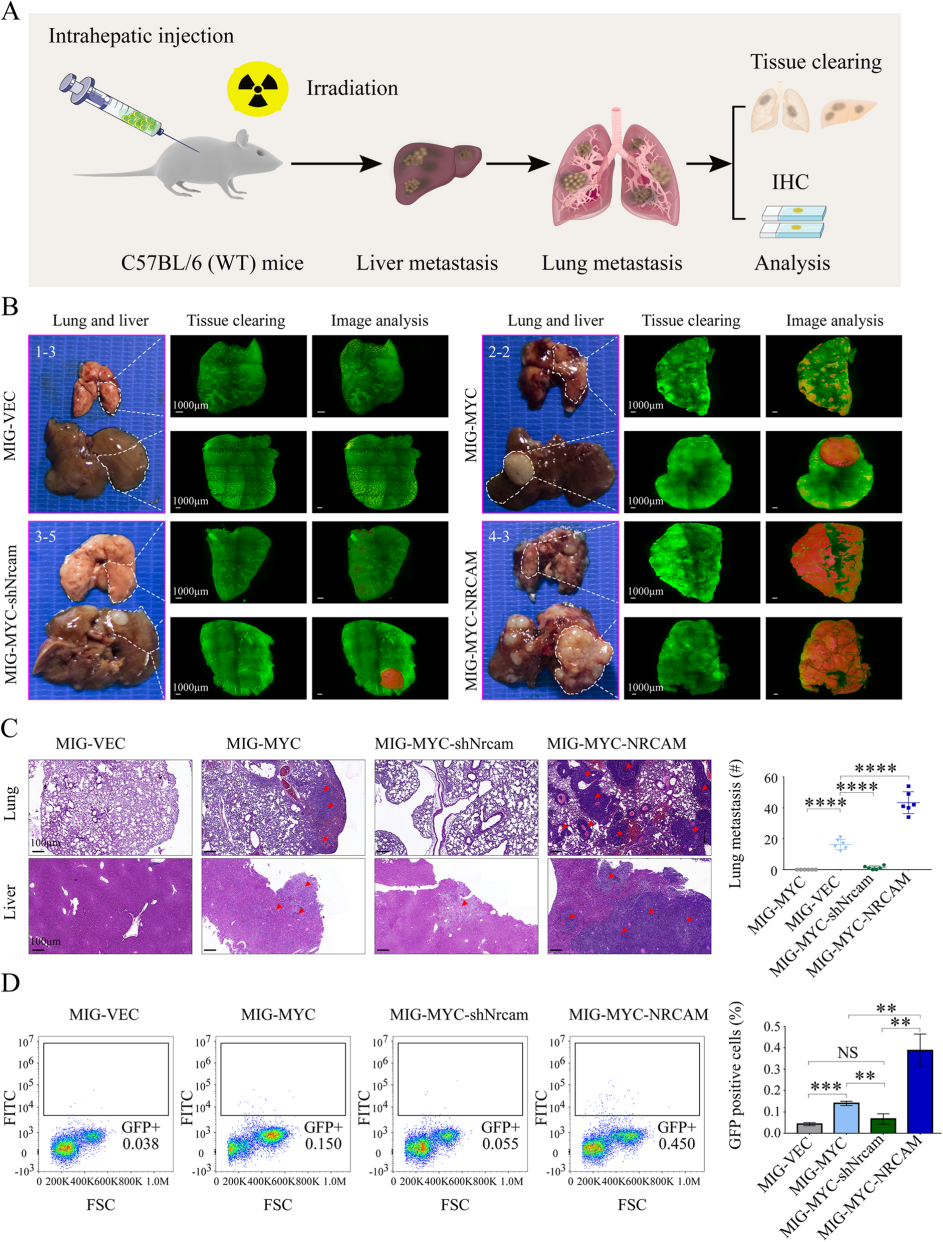
Effects of NRCAM expression on HCC metastasis activity in murine oncogene-driven allograft HCC model. **A** Murine oncogene-driven allograft HCC model and metastasis system workflow (6 mice in each group). **B** Tissue clearing and image analysis from representative MIG-Vec mice (Mouse: 1–3), MIG-MYC (Mouse: 2–2), MIG-MYC-shNrcam (Mouse: 3–5), and MIG-MYC-NRCAM (Mouse: 4–3) lung and liver: Mouse lung and liver tissue clearing assay (tumors are rendered red). **C** H&E staining for the MIG-Vec (Mouse: 1–3), MIG-MYC (Mouse: 2–2), MIG-MYC-shNrcam (Mouse: 3–5), and MIG-MYC-NRCAM (Mouse: 4–3) groups. Red arrows indicate tumor tissue in the lungs and livers. The number and size of HCC lung metastases in the MIG-Vec, MIG-MYC, MIG-MYC-shNrcam, and MIG-MYC-NRCAM groups were calculated. **D** LCSCs in mouse peripheral blood: GFP positive rates for the MIG-Vec, MIG-MYC, MIG-MYC-shNrcam, and MIG-MYC-NRCAM groups, blood was tested for three mice from each group. Data are represented as mean ± SD. *P* > 0.05 (NS), *P* < 0.01 (**), *P* < 0.001 (***), *P* < 0.0001 (****)

To further explore the effects of NRCAM on HCC and metastasis in vivo, MIG-Vec, MIG-MYC, MIG-MYC-shNrcam, and MIG-MYC-NRCAM organoids were transplanted through intrahepatic injection into C57BL/6 mice with transient irradiation induced immunodeficiency (Fig. S5A-C). The mice were sacrificed 28 days after allografting. One representative lung and liver were fixed, used for tissue clearing, and H&E stained (Fig. 6A). The tissue clearing and image analysis (Fig. 6B, Fig. S5D) demonstrated that there were 275 tumors within the lung from the MIG-MYC-NRCAM group (0.61/mm^3^), the tumor occupied 16.28% of the total lung, the most extensive tumor was 7.77mm^3^. Whereas the MIG-MYC group (0.38/mm^3^) lung contained 190 tumors that occupied 1.45% of the entire lung, the biggest tumor was 1.37mm^3^. There were no apparent differences for the lung tumors from the MIG-Vec and MIG-MYC-shNrcam groups (Additional files 2, 3, 4 and 5, Table S9). Similar differences were found for the liver analysis, where 487 tumors were observed from the MIG-MYC-NRCAM group (0.39/mm^3^); the tumors occupied 54.35% of the total liver, and the largest tumor was 101.57mm^3^. There were 130 tumors in the liver from the MIG-MYC group (0.26/mm^3^); the tumor occupied 21.72% of the total liver, and the biggest tumor was 107.23mm^3^. There was only one tumor (volume 27.33mm^3^) in the liver from the MIG-MYC-shNrcam group, which was wholly encapsulated within the liver, occupying 4.36% of the total liver. No obvious tumors were present within the liver from the MIG-Vec group (Additional files 6, 7, 8 and 9, Table S10).

H&E staining and IHC analysis verified liver and lung metastasis (Fig. S5E, F). More extensive intrahepatic metastases and metastatic lung nodules were observed with a greater frequency in the MIG-MYC-NRCAM group compared to the MIG-MYC group. Whereas fewer metastatic lung nodules were detected in the MIG-MYC-shNrcam group compared to the MIG-MYC group (*P* < 0.0001) (Fig. 6C). Additionally, a significantly higher rate of (GFP positive) LCSCs were observed in peripheral blood from MIG-MYC-NRCAM group mice (compared to MIG-MYC mice). On the other hand, relatively few LCSCs were detected in the MIG-Vec and MIG-MYC-shNrcam group mouse peripheral blood (Fig. 6D). These results, when taken together, suggest that NRCAM increases the ability of LCSCs to migrate, invade and cause blood metastasis in vivo.

### NRCAM activated the WNT/β-catenin signaling pathway, EMT, and MMP3/7/14 in LCSCs

**Fig. 7:**
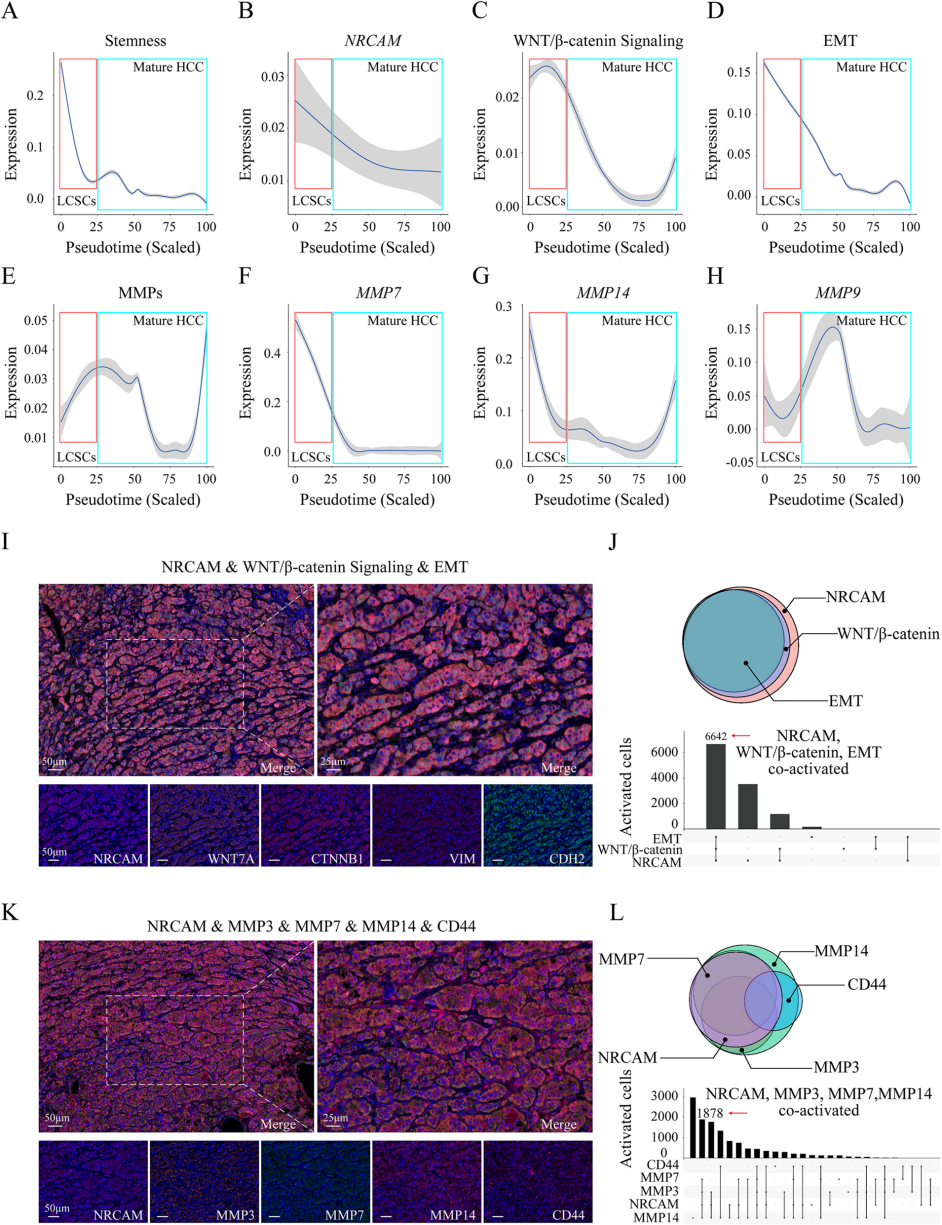
Identification of key NRCAM-mediated pathways that promote HCC metastasis. **A** Stemness, **B** *NRCAM* expression, **C** WNT/β-catenin signaling activity, **D** EMT activity, **E** MMPs expression, **F** *MMP7* expression, **G** *MMP14* expression and **H** *MMP9* expression change in pseudotime. **I** Multiplex immunofluorescent (mIF) mIF staining for NRCAM, WNT/β-catenin signaling, and EMT in metastatic HCC tissue. **J** Venn co-activation analysis for NRCAM, WNT/β-catenin signaling, and EMT in HCC cells. **K** mIF staining for NRCAM, MMP3, MMP7, MMP14 and CD44 in the metastatic HCC tumor tissue. **L** Venn co-activation analysis for MMP3, MMP7, MMP14, and CD44 in HCC cells

To explore pathways associated with NRCAM-mediated HCC metastasis, gene expression changes over pseudotime were observed using our scRNA-seq data set. LCSCs had higher stemness than mature HCC (Fig. 7A). *NRCAM* was highly expressed in LCSCs and decreased when LCSCs differentiated into mature HCC (Fig. 7B). Changes to *NRCAM* expression over pseudotime were consistent with changes to WNT/β-catenin pathway signaling (Fig. 7C) and EMT (Fig. 7D). MMPs were also observed to be changed in pseudotime (Fig. 7E), and are known to be closely associated with tumor metastasis. Specifically, *MMP7* changed consistently with *NRCAM* across pseudotime (Fig. 7F), *MMP14* expression was consistent with *NRCAM* in LCSCs but was reactivated in mature HCC (Fig. 7G), whereas *MMP9* expression did not appear to be associated with *NRCAM* (Fig. 7H). In the SRP278381 dataset, consistent results were observed; *MMP3* and *CD44* were also changed with *NRCAM* across pseudotime (Fig. S6).

Multiplex immunofluorescence analysis further confirmed that NRCAM, WNT7A, CTNNB1 (β-catenin), VIM (Vimentin), and CDH2 (N-Cadherin) were co-expressed in HCC-associated hepatocytes (Fig. 7I). Most HCC cells (6642 cells) with activated NRCAM also had WNT/β-catenin signaling pathway (WNT7A, CTNNB1 co-activated) and EMT (Vimentin and CDH2 co-activated) activation (Fig. 7J), which was consistent with the scRNA-seq results. Moreover, NRCAM was co-activated with MMP3, MMP7, and MMP14 in 1878 cells without CD44 expression (Fig. 7K, L). In addition, consistent results were observed in four other HCC tumor tissue layers (Fig. S7A, B) and some HCC adjacent normal layers (Fig. S7C, D). While the Notch signaling pathway was also activated in LCSCs (Figure S7E, F), multiplex immunofluorescence analysis confirmed that NRCAM and NOTCH1 were not co-activated in the same LCSCs (Fig. S7G). These findings indicated that NRCAM might play a vital role in activating the WNT/β-catenin signaling pathway and promote LCSCs metastasis through EMT and MMP3, MMP7, and MMP14 activation.

### NRCAM activated the β-catenin signaling pathway via MACF1 in LCSCs

**Fig. 8:**
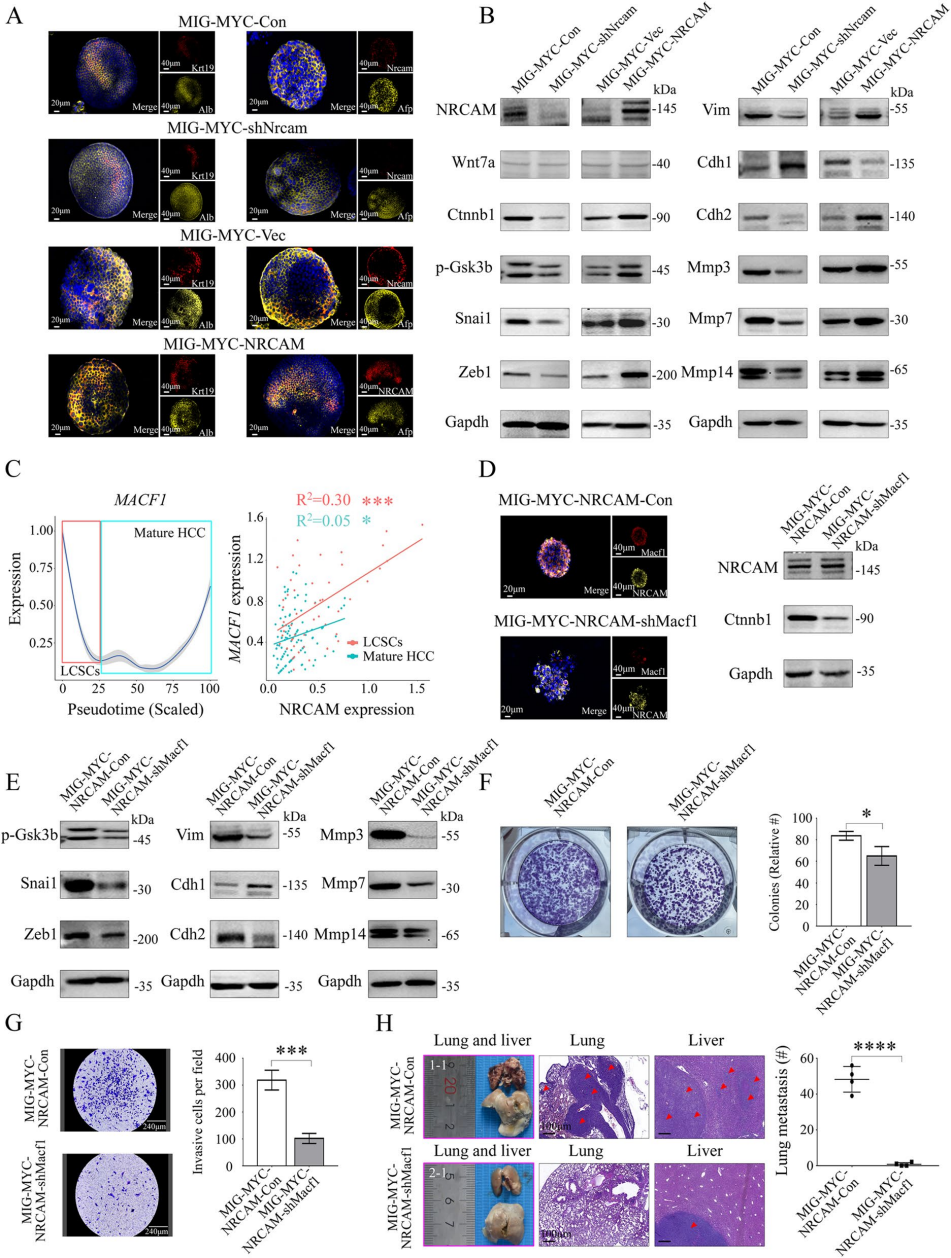
NRCAM activated the MACF1 mediated β-catenin signaling pathway to promote EMT and MMPs in LCSCs. **A** IF staining for Krt19, Alb, Nrcam, and Afp in MIG-Con, MIG-MYC-shNrcam, MIG-MYC-Vec, and MIG-MYC-NRCAM organoids. **B** Western blotting for key factors: Wnt/β-catenin signaling pathway, EMT, and metastasis MMPs in MIG-Con, MIG-MYC-shNrcam, MIG-MYC-Vec, and MIG-MYC-NRCAM organoids. **C** *MACF1* expression in pseudotime; *NRCAM* and *MACF1* expression correlation in LCSCs (R = 0.30, *P* < 0.001) and mature HCCs (R = 0.05, *P* < 0.05). **D** IF staining for NRCAM and Macf1 in MIG-MYC-NRCAM-Con and MIG-MYC-NRCAM-shMacf1 organoids. Western blotting for NRCAM and Ctnnb1 in MIG-MYC-NRCAM-Con and MIG-MYC-NRCAM-shMacf1 organoids. **E** Western blotting for key factors from the Wnt/β-catenin signaling pathway, EMT, and metastasis MMPs in MIG-MYC-NRCAM-Con and MIG-MYC-NRCAM-shMacf1 organoids. **F** Colony formation, **G** transwell assays for MIG-MYC-NRCAM-Con and MIG-MYC-NRCAM-shMacf1 organoids. **H** H&E staining for the MIG-MYC-NRCAM-Con (Mouse: 1–1) and MIG-MYC-NRCAM-shMacf1 (Mouse: 2–1) groups. Red arrows indicate tumor tissue in the lungs and livers. The number and size of HCC lung metastases in the MIG-MYC-NRCAM-Con and MIG-MYC-NRCAM-shMacf1 groups (4 mice in each group). *P* < 0.05 (*), *P* < 0.001 (***), *P* < 0.0001 (****)

The impact of NRCAM expression on the β-catenin signaling pathway, EMT, and MMPs activation, was explored using MIG-MYC-Con, MIG-MYC-shNrcam, MIG-MYC-Vec and MIG-MYC-NRCAM organoids (Fig. 8A). Western blotting was used to assess the level of key factors from the Wnt/β-catenin signaling pathway, EMT, and MMPs. The results verified that NRCAM might promote LCSC migration and invasion via the β-catenin signaling pathway by activating EMT and MMP3, MMP7, and MMP14 (Fig. 8B, Fig. S8A).

MACF1 is an important molecule that adjusts the invasive and metastatic potential of tumor cells*.* An interaction network analysis demonstrated that NRCAM and MACF1 had a “physical association” (Fig. S8B), suggesting that NRCAM and MACF1 were in close proximity. *MACF1* expression was consistent with *NRCAM* in LCSCs within the scRNA-seq dataset but not in mature HCC. A correlation analysis further confirmed that *MACF1* expression was associated with *NRCAM* in LCSCs (R = 0.30, *P* < 0.001) (Fig. 8C). A previous investigation [28] suggested that MACF1 can activate β-catenin signaling by enhancing CTNNB1 expression. Hence, Macf1 was knocked down in MIG-MYC-NRCAM organoids to determine whether NRCAM activated CTNNB1 via Macf1 (Fig. S8C). NRCAM and Macf1 were activated and co-located in MIG-MYC-NRCAM-Con organoids. The MIG-MYC-NRCAM-shMacf1 organoids displayed lower Ctnnb1 levels than the MIG-MYC-NRCAM-Con organoids (Fig. 8D). Meanwhile, Western blotting demonstrated that p-Gsk3b, Snai1, Zeb1, Cdh2, Vim, Mmp3, Mmp7, and Mmp14 were decreased in the MIG-MYC-NRCAM-shMacf1 organoids compared to MIG-MYC-NRCAM-Con organoids; whereas Cdh1 was increased (Fig. 8E, Fig. S8D). These results indicated that NRCAM activated the β-catenin signaling pathway via MACF1 in LCSCs.

A clone formation assay revealed that the MIG-MYC-NRCAM-shMacf1 organoids formed fewer colonies (Fig. 8F) when compared to the MIG-MYC-NRCAM-Con organoids (83.67 ± 4.04 vs. 65.00 ± 8.72, *P* = 0.028). In addition, a transwell assay indicated that MIG-MYC-NRCAM-shMacf1 organoid cells migrated at a significantly lower rate (Fig. 8G) when compared to the MIG-MYC-NRCAM-Con organoids (319 ± 37/field vs. 102 ± 19/field, *P* < 0.001). The in vivo tumor allografts (Fig. S8E) also revealed no obvious lung tumors and only one tumor in the liver from the MIG-MYC-NRCAM-shMacf1 group (Fig. 8H, Fig. S8F). These results indicated that MACF1 or β-catenin inhibitors downstream of NRCAM could be used to prevent LCSC metastasis.

## Discussion

High *NRCAM* expression was associated with HCC and a poorer survival probability in TCGA patients. Its expression was especially high in those with metastatic/advanced disease. These observations were further confirmed using an immunohistochemical analysis in tissue from HCC patients. NRCAM was expressed at high levels in the periportal area, which is associated with the presence of small oval cells. These cells are thought to be a source of LCSCs that participate in hepatocarcinogenesis and contribute towards a poorer prognosis [29].

Our study explored the diagnostic and prognostic utility of NRCAM not only using TCGA tumor expression profiles but also in serum. As such, our comparisons of NRCAM to AFP and PIVKA-II in patient blood samples better support the potential clinical utility of NRCAM. The cross-sectional study demonstrated that NRCAM may be useful as a prognostic biomarker. Prognostic biomarkers are not yet widely used in the clinic to guide the treatments given to HCC patients. Nevertheless, some notable studies have explored the potential utility of serum markers for this use, which produced the BALAD [30] and BALAD-2 [31] serological models. These models were designed to predict survival, which is helpful but may not be as informative as markers for a specific mechanism of poor prognosis that could enable the selection of personalized therapeutic schedules. In our study, NRCAM had the highest specificity of the three biomarkers (89.88%, versus 47.46% for AFP and 78.53% for PIVKA-II), NRCAM had a sensitivity of 47.46% (Table 5), which indicates that NRCAM may have utility for the prediction of a particular type of HCC. Although this notion requires further research, patients with elevated NRCAM might be more likely to have stem-cell type HCC. As NRCAM can be easily tested in serum, it may reduce the need for a liver biopsy. Hence, NRCAM could be clinically valuable for predicting metastasis in patients with early-stage HCC and for those likely to experience early metastasis, perhaps in combination with other markers. It is also worth noting that as a cross-sectional study, the utility of NRCAM and the biomarkers combinations for detecting metastatic/stem-cell type disease would ultimately require further confirmation via a multi-center prospective study.

The applicability of NRCAM to stem cell type disease is supported by the single-cell sequencing data. *NRCAM* was exceptionally high in LCSCs and was closely associated with WNT/β-catenin signaling pathway gene expression but not the NOTCH pathway. Additionally, *NRCAM* was significantly elevated and correlated with *MYC* in LCSCs, the rho depicted a moderate association, and their expression was not correlated in mature HCC cells. The correlation of NRCAM [14, 15] and MYC [32] expression is supported by their association with WNT/β-catenin signaling. Indeed, Conacci-Sorrell et al. [14] noted that *NRCAM* and *MYC* were induced by plakoglobin (γ-catenin), which plays a role in WNT signaling that is distinct from β-catenin [33]. The immunofluorescence and western blotting results (Fig. 8A, Fig. S4F, J) in our research indicated that *MYC* overexpression in LCSCs increased NRCAM; while *NRCAM* overexpression did not induce MYC. These results also demonstrated that MYC activated NRCAM in LCSCs.

The source of LCSCs remains controversial [34]. Some studies consider that LCSCs are dedifferentiated mature HCC cells, while others suggest that LCSCs are generated by a genomic mutation in liver stem cells [35, 36]. Our study supports the notion that LCSCs are derived via the malignant transition of liver stem cells and that activation of specific genes, such as *MYC* and *NRCAM,* leads to a significantly increased risk of a metastatic phenotype. The in vitro and in vivo models utilized MYC to induce liver progenitor cells and produce liver cancer stem cells. Notably, the modulation of NRCAM in these cells was associated with metastatic phenotypes. The tissue-clearing assay clarified that the larger liver tumors found in the MIG-MYC-NRCAM were, in fact, made up of many small tumors, which is consistent with metastatic phenotypes. It is interesting that tumor-bearing mice with elevated NRCAM presented with a significantly greater cancer cell number in their peripheral blood and significantly less when *Nrcam* was knocked down (Fig. 6D). Therefore, it appears that NRCAM facilitates HCC metastasis to distant tissues by enhancing the ability of LCSCs to escape from tumors into the bloodstream.

NRCAM overexpression has previously been associated with vascular invasion in colorectal cancer [37, 38]. It is notable that our scRNAseq data revealed that NRCAM was highly expressed in endothelial cells, mirroring findings from an in vitro angiogenesis model where NRCAM was also identified in endothelial cells [39]. Therefore, NRCAM might contribute to vascular invasion in HCC and, thereby, metastasis. However, our in vivo models only explored NRCAM expression in cancer cells; it may be interesting for future studies to explore the metastatic contribution of NRCAM in blood vessel-associated cells. It is also notable that MMPs were identified to be differentially expressed in response to NRCAM, which may be induced by the NRCAM WNT/β-catenin positive feedback loop described by Zhang et al. (2017). MMPs have links to metastasis and vascular invasion; however, clinical trials using broad-spectrum MMP inhibitors have been unsuccessful due to adverse effects associated with their use. Nevertheless, targeting specific MMPs may prove to be efficacious and safe [40]. Our study demonstrated that NRCAM might offer a pathway to safely indirectly target MMP3, MMP7, and MMP14 and influence their activities. Therefore, it may be worthwhile to explore further the interactions between NRCAM and MMP3, MMP7, and MMP14.

While NRCAM has been described as affecting WNT/β-catenin signaling in other diseases (including various malignancies), the mechanism by which this occurred was not fully understood. Our scRNAseq data indicated that *MACF1* expression was closely related to NRCAM in LCSCs, which has previously been suggested to increase osteoblast proliferation by enhancing β-catenin signaling and *CTNNB1* expression [41]. We further demonstrated in vivo that NRCAM directly activated β-catenin via MACF1 without activating WNT. Moreover, MACF1 inhibition significantly blocked the effects associated with NRCAM activation on β-catenin signaling and HCC metastasis. Hence, further studies on compounds targeting MACF1 and NRCAM are warranted for β-catenin pathway mediated LCSC inhibition. We identified two important features regarding the NRCAM/WNT/β-catenin axis: 1) in vivo NRCAM knockdown fully blocked LCSC-mediated metastasis but did not completely stop the formation of HCC by LCSCs (Fig. 6B). 2) NRCAM directly activated β-catenin through MACF1 without activating WNT. This is particularly relevant for clinical strategies since β-catenin pathway inhibitors (MACF1 or β-catenin/TCF4 inhibitors downstream of NRCAM) will not prevent LCSC metastasis via WNT. This suggests that β-catenin pathway inhibitors may better treat HCC when used in combination with an in situ treatment (TACE or hepatectomy).

## Conclusion

This study supports the exploration of NRCAM as a novel biomarker representing metastatic LCSC activation in HCC. Moreover, NRCAM could be valuable for patients predisposed to metastatic HCC, enabling early diagnosis and treatment selection. We also show that NRCAM potentiates LCSC dissemination and metastasis via MACF1 mediated β-catenin signaling. Ultimately, targeting NRCAM, MACF1, or β-catenin could provide a powerful means to prevent LCSC-based metastasis.

### Supplementary Information

**Additional file 1. **Baseline characteristics of patients.

**Additional file 2: Movie S1.** Mouse 1-3 Lung.

**Additional file 3: Movie S2.** Mouse 2-2 Lung.

**Additional file 4: Movie S3.** Mouse 3-5 Lung.

**Additional file 5: Movie S4.** Mouse 4-3 Lung.

**Additional file 6: Movie S5.** Mouse 1-3 Liver.

**Additional file 7: Movie S6.** Mouse 2-2 Liver.

**Additional file 8: Movie S7.** Mouse 3-5 Liver.

**Additional file 9: Movie S8.** Mouse 4-3 Liver.

**Additional file 10:** Supplementary Materials and Methods. Supplementary Text. **Fig. S1.** The use of NRCAM for HCC diagnosis and metastasis prediction. (A) Flowchart. HCC: Hepatocellular carcinoma, ICC: Intrahepatic cholangiocarcinoma, TCGA: Cancer Genome Atlas, ROC: receiver operating curve. (B) A ROC was used to evaluate the predictive value of serum AFP, PIVKA-II, and NRCAM for HCC diagnosis. (C) IHC staining for ARG1 and Hep Par 1 in HCC without metastasis. (D) IHC staining of ARG1 and Hep Par 1 for HCC with metastasis. **Fig. S2.** Single-cell profile and evolution trajectory of metastasis HCC. (A) Experiment procedure overview. Five scRNA-seq datasets were generated from HCC tumor tissues to provide transcriptomes for 36,085 individual cells. H&E, IHC, and IF were performed on FFPE tissue in parallel. (B) A UMAP demonstrating the 23 main cell clusters. (C) Violin plots showing the normalized expression for cell type-specific markers. (D) Cell type assignment to clusters. (E) Potential hepatocyte trajectory with color scales representing pseudotime. *Epcam* expression across the trajectory with color scales representing expression. Potential hepatocyte trajectory for seven distinct cell states. State 1-2 represented LCSCs, and state 3-7 mature HCC. The red arrows demonstrate the potential cell-level evolutionary trajectory. **Fig. S3.** Marker expression in HCC. (A) KRT19 expression trajectory, color scale represents expression. (B) trajectory of adult liver markers (TAT, TDO2, SSTR2, CYP7A1, CYP3A4 and CYP2B6), color scale represents expression. (C) large-scale copy number variation (CNV) identified in hepatocytes from the five patients using scRNA-seq data, normal hepatocytes were used as a reference; amplification is represented using red and blue for deletion. (D) Cell ratio of aneuploid in clusters. (E) Epithelial scores (consisting of SFN, EPCAM, KRT17, KRT86, KRT81, KRT18, KRT222, KRT10, KRT23, KRT19, KRT80, KRT36, KRT17 and KRT27) for the hepatocyte clusters. (F) UMAP demonstrating malignant cells (red) identified by CNV and epithelial scoring. (G) GSEA plots showing stemness gene enrichment patterns and marker expression according to *NRCAM* status (high to low) in TCGA samples. (H) *MYC* expression according to *NRCAM* status (high to low) in TCGA samples. (I) TCGA *NRCAM* and *MYC* expression correlation. *P*=<0.05 (*). **Fig. S4.** Regulation of NRCAM in mouse LCSCs. (A) MIG-Vec, MIG-MYC, MIG-NRCAM, MIG-MYC-NRCAM, and MIG-MYC-shNRCAM plasmid structures. (B) qPCR and (C) western blotting for shRNA-mediated NRCAM inhibition, shRNA1 had a good inhibitory effect on NRCAM and was used in subsequent experiments. (D, E) The GFP positive rate of MIG-Vec and MIG-MYC organoids two and 14 days after transduction. (F) MYC and Nrcam levels in MIG-Vec and MIG-MYC organoids. (G) Plate clone formation assay for MIG-Vec and MIG-MYC organoids. (H, I) GFP positive rate of MIG-Vec and MIG-NRCAM organoids two and 14 days after transduction. (J) NRCAM and Myc levels in MIG-Vec and MIG-NRCAM organoids. (K) plate clone formation assay. The data was displayed using the mean ± SD where applicable. *P*=<0.05 (*), *P*=<0.01 (**), *P*=<0.001 (***). **Fig. S5.** Identification of HCC metastasis in the murine oncogene-driven HCC allograft model. (A) MYC levels in MIG-MYC, MIG-MYC-NRCAM, and MIG-MYC-shNRCAM organoids. (B) Intrahepatic injection of MIG-Vec, MIG-MYC, MIG-MYC-NRCAM, and MIG-MYC-shNRCAM organoids into irradiation-induced transient immune deficient C57BL/6 mice. (C) Mouse lungs and livers 28 days after allografting for the MIG-Vec, MIG-MYC, MIG-MYC-shNRCAM, and MIG-MYC-NRCAM groups. (D) Tissue clearing and image analysis for lung and liver from the MIG-Vec (Mouse: 1-3), MIG-MYC (Mouse: 2-2), MIG-MYC-shNrcam (Mouse: 3-5), and MIG-MYC-NRCAM (Mouse: 4-3) group mice. (E) HE staining for the MIG-Vec (Mouse: 1-3), MIG-MYC (Mouse: 2-2), MIG-MYC-shNrcam (Mouse: 3-5) and MIG-MYC-NRCAM (Mouse: 4-3) groups. (F) IHC staining for NRCAM and Hep Par 1 in the lung and liver from MIG-MYC-NRCAM (Mouse: 4-3) group mice. The data was represented using the mean ± SD where applicable, *P*=>0.05 (NS). **Fig. S6.** SRP278381 GEO database single-cell profile and evolution trajectory. (A) tSNE displaying the 21 main cell clusters and cell types, a differential gene expression analysis was used to identify cluster-specific markers. (B) *NRCAM* is predominately expressed in cluster 17, a group of bi-potent stem cells (KRT19+ and EPCAM+). (C) tSNE displaying malignant cells (red, identified using epithelial score), NRCAM+ bi-potent stem cells were malignant, and could be defined as LCSCs. NRCAM+ bi-potent stem cell distribution (Cluster 17) was mostly in adjacent normal tissue (adjacent normal: 592 from 629). (D) Hepatocyte trajectory analysis (arrow displays potential evolutionary direction): the potential trajectory of all hepatocytes, the color scale represents pseudotime; seven distinct cell states (colored by state 1-7) were identified across the potential hepatocyte trajectory. Liver stem cells were present in state 1, mature hepatocytes in state 2-6, and HCC cells in state 7. (E) Epithelial score genes, *NRCAM,* and *MYC* expression in pseudotime. (F) WNT/β-Catenin signaling activity, EMT activity, *MMP3,* *MMP7, MMP14, *and* CD44 *expression in pseudotime. **Fig. S7.** Identification of the key pathway associated with NRCAM activation in metastatic HCC. (A) Multiplex immunofluorescent (mIF) staining for NRCAM, Wnt/β-catenin signaling, and EMT in layers 2-5. Venn diagram displaying NRCAM, Wnt/β-catenin signaling, and EMT co-activation in HCC cells. (B) mIF staining for NRCAM, MMP3, MMP7, MMP14 and CD44. Venn diagram displaying MMP3, MMP7, MMP14, and CD44 co-activation in HCC cells. (C) mIF staining for NRCAM, Wnt/β-catenin signaling, and EMT in layer 1-5. Venn diagram displaying NRCAM, Wnt/β-catenin signaling, and EMT co-activation in HCC cells. (D) mIF staining for NRCAM, MMP3, MMP7, MMP14 and CD44. Venn diagram displaying MMP3, MMP7, MMP14, and CD44 co-activation in HCC cells. (E) Notch signaling activity on the trajectory (color scale representing activity level). (F) Notch signaling activity changes in pseudotime. (G) mIF staining for NRCAM and NOTCH1 in metastatic HCC tumor tissue demonstrates few cells with NRCAM and NOTCH1 co-activation. **Fig. S8.** NRCAM, key factors in MACF1 mediated β-catenin signaling pathway, EMT and MMPs expression in LCSCs. (A) NRCAM, Wnt7a, Ctnnb1, p-Gsk3b, Snai1, Zeb1, Vim, Cdh1, Cdh2, Mmp3, Mmp7 and Mmp14 levels. (B) Interaction Network analysis of NRCAM and MACF1 from IntAct (https://www.ebi.ac.uk/intact/home). (C) shRNA inhibition of MACF1. The shRNA2 had a good inhibitory effect on MACF1 and was used in subsequent experiments. (D) NRCAM, Ctnnb1, p-Gsk3b, Snai1, Zeb1, Vim, Cdh1, Cdh2, Mmp3, Mmp7 and Mmp14 levels. (E) Mouse lungs and livers 28 days after allografting for the MIG-MYC-NRCAM-Con and MIG-MYC-NRCAM-shMacf1 groups. (F) HE staining for the MIG-MYC-NRCAM-Con (Mouse: 1-1) and MIG-MYC-NRCAM-shMacf1 (Mouse: 2-1) groups. Where applicable, the data was represented using mean ± SD (*n*=3). *P*=<0.05 (*), *P*=<0.01 (**), *P*=<0.001 (***), *P*=<0.0001 (****). **Table S1.** Clinicopathological information for the two PDX HCC models. **Table S2.** Clinicopathological information for five patients with HCC metastasis. T**able S3.** shRNAs targeting Nrcam and Macf1. **Table S4.** The qPCR primer sequences. **Table S5.** Antibodies. **Table S6.** NrCAM mRNA in TCGA liver cancer and normal tissues. **Table S7.** HCC diagnostic ROCs for AFP, PIVKA-II and NRCAM. **Table S8.** Univariate and multivariate logistic analysis in HCC diagnosis. **Table S9.** Pulmonary metastasis associated with different LCSCs. **Table S10.** Intrahepatic metastasis associated with different LCSCs.

## Data Availability

The raw sequencing data from this study was deposited in the BIG Data Center Genome Sequence Archive, Beijing Institute of Genomics, Chinese Academy of Sciences: https://ngdc.cncb.ac.cn/gsa-human/s/1camqBW8, https://ngdc.cncb.ac.cn/gsa-human/s/TwWFB3Z0. The original experimental data from this study was deposited in the Harvard Dataverse: https://doi.org/10.7910/DVN/XU878V.

## Abbreviations

LCSCs: Liver cancer stem cells
NRCAM: Neuronal cell adhesion molecule
EMT: Epithelial-mesenchymal transition
AFP: Alpha-fetoprotein
MMPs: Matrix metalloproteinases
scRNAseq: Single-cell RNA sequencing
TCGA: The cancer genome atlas
PIVKA-II: Protein induced by vitamin K absence/antagonist- II
ROC: Receiver operating characteristic
AUC: Area Under Curve
