# Supplementary material for: Liver cancer stem cell dissemination and metastasis: uncovering the role of NRCAM in hepatocellular carcinoma
Source: J Exp Clin Cancer Res. 2023 Nov 22;42:311. doi: 10.1186/s13046-023-02893-w (PMC10664624; doi:10.1186/s13046-023-02893-w)
Supplement: Supplementary file 10 — Additional file 10: Supplementary Materials and Methods. Supplementary Text. Fig. S1. The use of NRCAM for HCC diagnosis and metastasis prediction. (A) Flowchart. HCC: Hepatocellular carcinoma, ICC: Intrahepatic cholangiocarcinoma, TCGA: Cancer Genome Atlas, ROC: receiver operating curve. (B) A ROC was used to evaluate the predictive value of serum AFP, PIVKA-II, and NRCAM for HCC diagnosis. (C) IHC staining for ARG1 and Hep Par 1 in HCC without metastasis. (D) IHC staining of ARG1 and Hep Par 1 for HCC with metastasis. Fig. S2. Single-cell profile and evolution trajectory of metastasis HCC. (A) Experiment procedure overview. Five scRNA-seq datasets were generated from HCC tumor tissues to provide transcriptomes for 36,085 individual cells. H&E, IHC, and IF were performed on FFPE tissue in parallel. (B) A UMAP demonstrating the 23 main cell clusters. (C) Violin plots showing the normalized expression for cell type-specific markers. (D) Cell type assignment to clusters. (E) Potential hepatocyte trajectory with color scales representing pseudotime. Epcam expression across the trajectory with color scales representing expression. Potential hepatocyte trajectory for seven distinct cell states. State 1-2 represented LCSCs, and state 3-7 mature HCC. The red arrows demonstrate the potential cell-level evolutionary trajectory. Fig. S3. Marker expression in HCC. (A) KRT19 expression trajectory, color scale represents expression. (B) trajectory of adult liver markers (TAT, TDO2, SSTR2, CYP7A1, CYP3A4 and CYP2B6), color scale represents expression. (C) large-scale copy number variation (CNV) identified in hepatocytes from the five patients using scRNA-seq data, normal hepatocytes were used as a reference; amplification is represented using red and blue for deletion. (D) Cell ratio of aneuploid in clusters. (E) Epithelial scores (consisting of SFN, EPCAM, KRT17, KRT86, KRT81, KRT18, KRT222, KRT10, KRT23, KRT19, KRT80, KRT36, KRT17 and KRT27) for the hepatocyte clusters. (F) UMA [file 13046_2023_2893_MOESM10_ESM.docx]

Supplementary Materials

**Liver cancer stem cell dissemination and metastasis: Uncovering the role of NRCAM in HCC**

Lingyun Zhou, Linye He, Chang-Hai Liu, Huandi Qiu, Li Zheng, Klarke Michael Sample, Qin Wu, Jiaxin Li, Kunlin Xie, Javier Ampuero, Zhihui Li, Duoduo Lv, Miao Liu, Manuel Romero-Gómez, Yiguo Hu, Hong Tang

This PDF file includes:

Supplementary Materials and Methods

Supplementary Text

Supplementary Figures. S1 to S8

Supplementary Tables S1 to S10

Supplementary References

## Supplementary Materials and Methods

### 1. Single-cell RNA-seq library construction and sequencing

Single-cell RNA-Seq libraries were prepared using Chromium Next GEM Single Cell 3ʹ Reagent Kits v3.1 (10×Genomics). Briefly, an appropriate number of cells were mixed with reverse transcription reagent and then loaded to the sample well in Chromium Next GEM Chip G. Subsequently, Gel Beads and Partitioning Oil were dispensed into corresponding wells separately in the chip. After emulsion droplet generation, reverse transcription was performed at 53℃ for 45 minutes and inactivated at 85℃ for five minutes. Next, cDNA was purified from a broken droplet and amplified in the PCR reaction. The amplified cDNA product was then cleaned, fragmented, end-repaired, A-tailed, and ligated to the sequencing adaptor. Finally, indexed PCR was performed to amplify genes containing a 3’ poly A tail and add a Cell Bar code and Unique Molecular Index. The indexed sequencing libraries were cleaned with SPRI beads, quantified by quantitative PCR (KAPA Biosystems KK4824), and sequenced on an Illumina NovaSeq 6000 as paired ends with a 150bp read length.

### 2. Single-cell bioinformatics analysis

**Sequencing data quality control**

Fastp (v0.20.1)^(1)^ was used to trim primer sequences and low quality bases of raw reads and collect the basic statistics. The specific parameters are summarized below: 1) A 4 bp sliding window was moved from the front (5') to the tail. Once the mean quality of the bases in the window was below 10, the bases in the window, along with the subsequent bases, would be dropped, and the analysis within the read was finished. The leading N bases were also trimmed. 2) A 1 bp sliding window was moved from the tail (3') to the front. The bases in the window were dropped if their mean quality was below 3, and the window kept moving until the last base. The trailing N bases were also trimmed, similar to the Trimmomatic TRAILING method. 3) The auto adapter was detected for PE data. 4) The trimmed Reads shorter than 60 bp were discarded. The cleaned reads after trimming were used in the following steps.

**Processing the single-cell RNA sequencing data**

The Cell Ranger^(2)^ Single-Cell Software Suite was used to perform sample demultiplexing, barcode processing, and single-cell 3’ gene counting (<http://software.10xgenomics.com/single-cell/overview/welcome>). First, UMI tags and barcode sequences were extracted from Read1. Then, Read2, which contains the cDNA insert, was aligned to an appropriate reference genome using STAR^(3)^. Next, barcodes and UMIs were filtered. All known barcodes that are one Hamming distance away from an observed barcode are considered. Then, the posterior probability that a sequencing error produced the observed barcode is computed, given the base qualities of the observed barcode and the prior probability of observing the candidate barcode (taken from the overall barcode count distribution). If the posterior probability for any candidate barcode is at least 0.975, then the barcode is corrected to the candidate barcode with the highest posterior probability. If all candidate sequences are equally probable, then the one appearing first by lexical order is picked. UMIs with sequencing quality score>10 were considered valid if they were not homopolymers. Qual=10 implies 90% base call accuracy. A UMI that is 1-Hamming-distance away from another UMI (with more reads) for the same cell barcode and gene is corrected to the UMI with more reads. This approach is nearly identical to that described by Jaitin *et al.* ^(4)^ and is similar to Klein *et al.* ^(5)^. Last, PCR duplicates were marked if two sets of read pairs shared a barcode sequence, a UMI tag, and a gene ID. Only confidently mapped (MAPQ=255) non-PCR duplicates with valid barcodes and UMIs were used to generate a gene-barcode matrix. Cell barcodes were determined based on the distribution of UMI counts, which had two key steps: 1) The original Cell Ranger cell calling algorithm is used to identify the primary mode of high RNA content cells, using a cutoff based on the total UMI count for each barcode. Cell Ranger takes as input the expected number of recovered cells, N (see-expect-cells). Where m is set to the 99th percentile of the top N barcodes by total UMI counts --all barcodes whose total UMI counts exceed m/10 are called as cells in the first pass. 2) Then, a set of barcodes with low UMI counts that likely represent ‘empty’ GEM partitions is selected. A model of the RNA profile of selected barcodes is created. This model, called the background model, is a multinomial distribution over genes. It uses Simple Good-Turing smoothing to provide a non-zero model estimate for genes that were not observed in the representative empty GEM set. Finally, the RNA profile of each barcode not called as a cell in the first step is compared to the background model. Barcodes whose RNA profile strongly disagrees with the background model are added to the set of positive cell calls. This second step identifies cells that are clearly distinguishable from the profile of empty GEMs, even though they may have much lower RNA content than the largest cells in the experiment. The number of reads that provide meaningful information is calculated as the product of four metrics: (1) valid barcodes, (2) valid UMI, (3) associated with a cell barcode, and (4) confidently mapped to exons.

## Supplementary Text

### 1. Elevated NRCAM serum levels are linked to HCC metastasis

Elevated NRCAM serum level was determined to be an independent predictor for HCC diagnosis after adjusting for gender, HBV infection status, liver cirrhosis, platelet count, ALT, AST, and AFP (Table S7). Elevated serum NRCAM levels also independently predicted metastasis in HCC patients after adjusting for age, detectable HBV DNA viral load, tumor size, the number of nodules, tumor differentiation, vascular invasion, BCLC stage, platelet count, Albumin and PIVKA-II (Table 4). Interestingly, the optimal NRCAM serum level threshold for HCC metastasis (2074.21 pg/ml) was almost twice the level of the primary HCC threshold (1091.93 pg/ml) (Table 5). This suggests that elevated NRCAM serum levels are linked to HCC metastasis.

### 2. NRCAM expression was analyzed in the SRP278381 dataset

*NRCAM* expression was analyzed in the SRP278381 dataset from the Gene Expression Omnibus (GEO) database (Fig.S6A). Cells expressing *NRCAM* were predominately expressed within cluster 17, a group of bi-potent steam cells (KRT19+ and EPCAM+) (Fig.S6B). Epithelial scoring showed that the NRCAM+ bi-potent steam cells were malignant, defined as LCSCs, and were mostly present in adjacent normal tissue (Fig.S6C). These results indicate that NRCAM-activated LCSCs can be located within normal tissue adjacent to HCC, which could be associated with early HCC metastasis. A pseudotime analysis demonstrated a liver stem cell to hepatocytes to HCC trajectory (Fig.S6D). HCC malignant cell trajectory was consistent with increased expression of *NRCAM* and *MYC* (Fig.S6E). Changes to WNT/β-catenin signaling, EMT, *MMP3*, *MMP7*, *MMP14,* and *CD44* (Fig.S6F) were consistent with *NRCAM* in pseudotime.

### 3. NRCAM activates EMT and MMP3, 7, 14 in LCSCs via MACF1 mediated β-catenin signaling pathway

The impact of NRCAM expression on the β-catenin signaling pathway, EMT, and MMPs activation was explored using MIG-MYC-Con, MIG-MYC-shNrcam, MIG-MYC-Vec and MIG-MYC-NRCAM organoids (Fig.8A). Western blotting was used to assess the level of key factors from the Wnt/β-catenin signaling pathway. Ctnnb1, p-Gsk3b, Snai1, and Zeb1 were decreased in MIG-MYC-shNrcam organoids compared to MIG-MYC-Con organoids. Likewise, the level of Ctnnb1 and p-Gsk3b was increased in MIG-MYC-NRCAM organoids, along with Snai1 and Zeb1. Western blotting was also used to assess MMP levels and key factors associated with EMT in the MIG-MYC-Con, MIG-MYC-shNrcam, MIG-MYC-Vec, and MIG-MYC-NRCAM organoids. Cdh1 was increased in MIG-MYC-shNrcam organoids compared to MIG-MYC-Con organoids, whereas Cdh2 and Vim were decreased. Consistently, Cdh1 was reduced in MIG-MYC-NRCAM organoids compared to MIG-MYC-Vec organoids, while Cdh2 and Vim were increased. Mmp3, Mmp7, and Mmp14 levels were decreased in MIG-MYC-shNrcam organoids and increased in MIG-MYC-NRCAM organoids (Fig.S8D). These results verified that NRCAM may promote LCSC migration and invasion via the β-catenin signaling pathway by activating EMT and MMP3, MMP7, and MMP14.

## Supplementary Figures

**
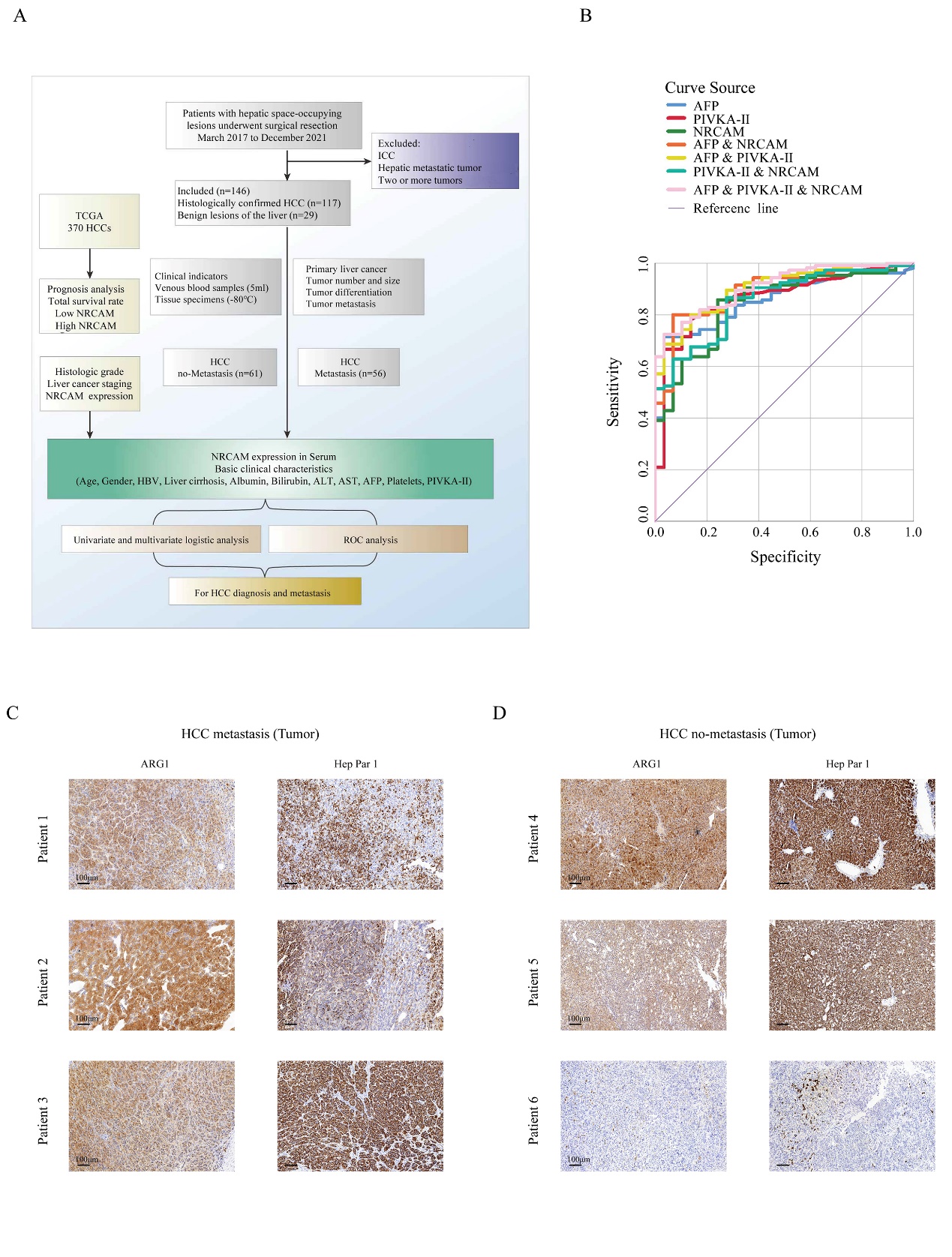
**

**Fig. S1. The use of NRCAM for HCC diagnosis and metastasis prediction.**

**(A)** Flowchart. HCC: Hepatocellular carcinoma, ICC: Intrahepatic cholangiocarcinoma, TCGA: Cancer Genome Atlas, ROC: receiver operating curve. **(B)** A ROC was used to evaluate the predictive value of serum AFP, PIVKA-II, and NRCAM for HCC diagnosis. **(C)** IHC staining for ARG1 and Hep Par 1 in HCC without metastasis. **(D)** IHC staining of ARG1 and Hep Par 1 for HCC with metastasis.

**
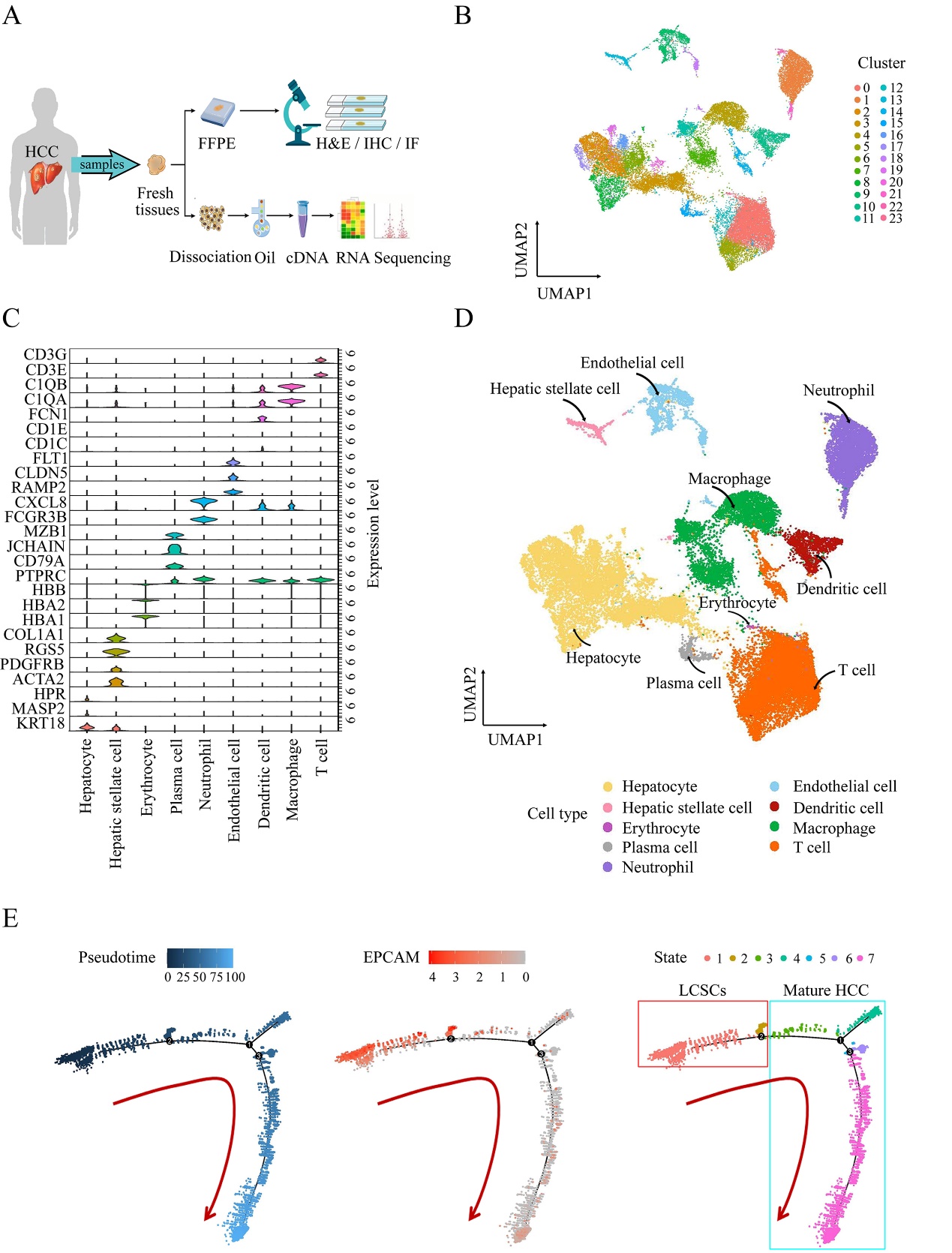
**

**Fig.S2. Single-cell profile and evolution trajectory of metastasis HCC.**

**(A)** Experiment procedure overview. Five scRNA-seq datasets were generated from HCC tumor tissues to provide transcriptomes for 36,085 individual cells. H&E, IHC, and IF were performed on FFPE tissue in parallel. **(B)** A UMAP demonstrating the 23 main cell clusters. **(C)** Violin plots showing the normalized expression for cell type-specific markers. **(D)** Cell type assignment to clusters. **(E)** Potential hepatocyte trajectory with color scales representing pseudotime. *Epcam* expression across the trajectory with color scales representing expression. Potential hepatocyte trajectory for seven distinct cell states. State 1-2 represented LCSCs, and state 3-7 mature HCC. The red arrows demonstrate the potential cell-level evolutionary trajectory.

**
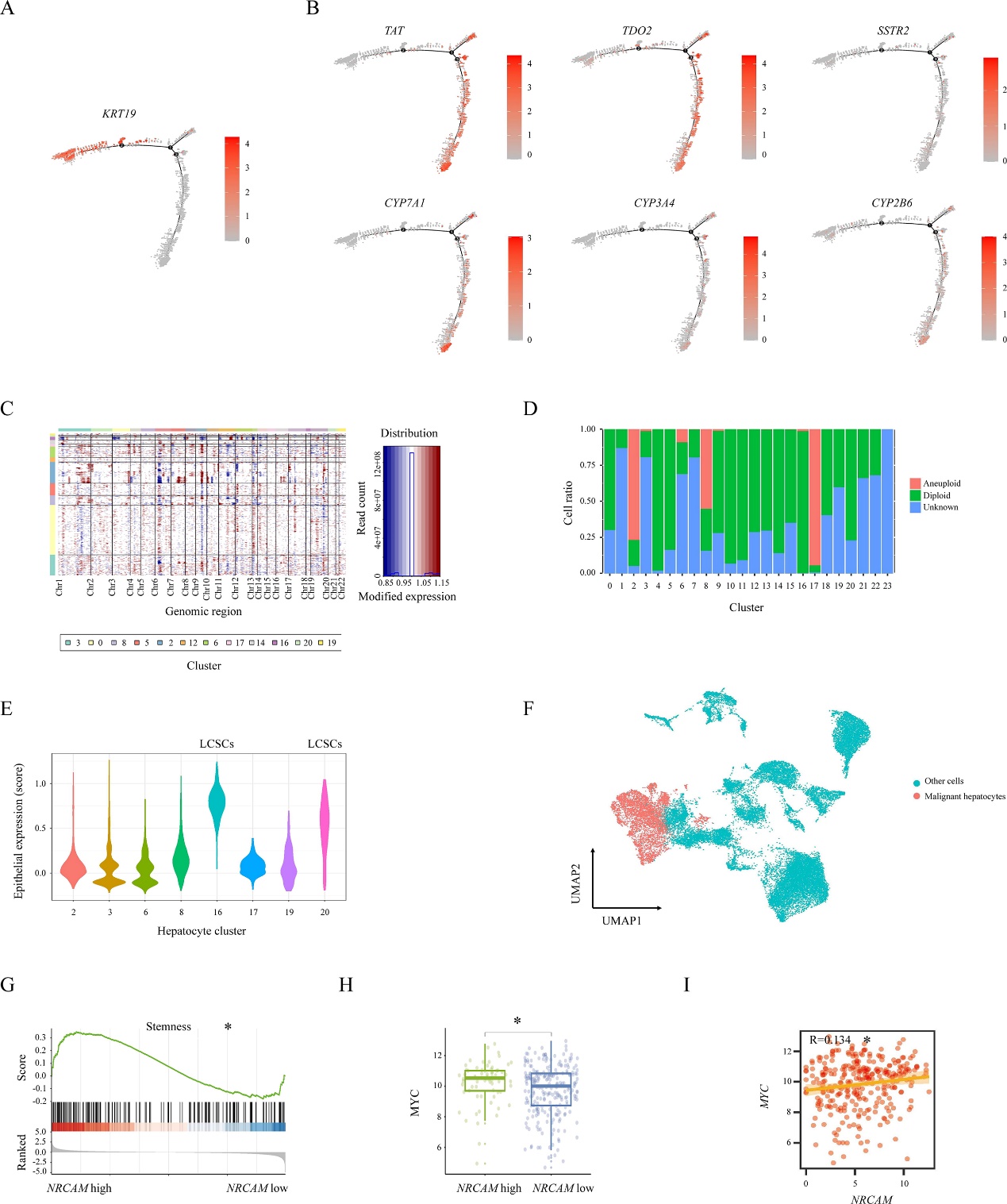
**

**Fig. S3. Marker expression in HCC.**

**(A)** KRT19 expression trajectory, color scale represents expression. **(B)** trajectory of adult liver markers (TAT, TDO2, SSTR2, CYP7A1, CYP3A4 and CYP2B6), color scale represents expression. **(C)** large-scale copy number variation (CNV) identified in hepatocytes from the five patients using scRNA-seq data, normal hepatocytes were used as a reference; amplification is represented using red and blue for deletion. **(D)** Cell ratio of aneuploid in clusters. **(E)** Epithelial scores (consisting of SFN, EPCAM, KRT17, KRT86, KRT81, KRT18, KRT222, KRT10, KRT23, KRT19, KRT80, KRT36, KRT17 and KRT27) for the hepatocyte clusters. **(F)** UMAP demonstrating malignant cells (red) identified by CNV and epithelial scoring. **(G)** GSEA plots showing stemness gene enrichment patterns and marker expression according to *NRCAM* status (high to low) in TCGA samples. **(H)** *MYC* expression according to *NRCAM* status (high to low) in TCGA samples. **(I)** TCGA *NRCAM* and *MYC* expression correlation. P=<0.05 (*).

**
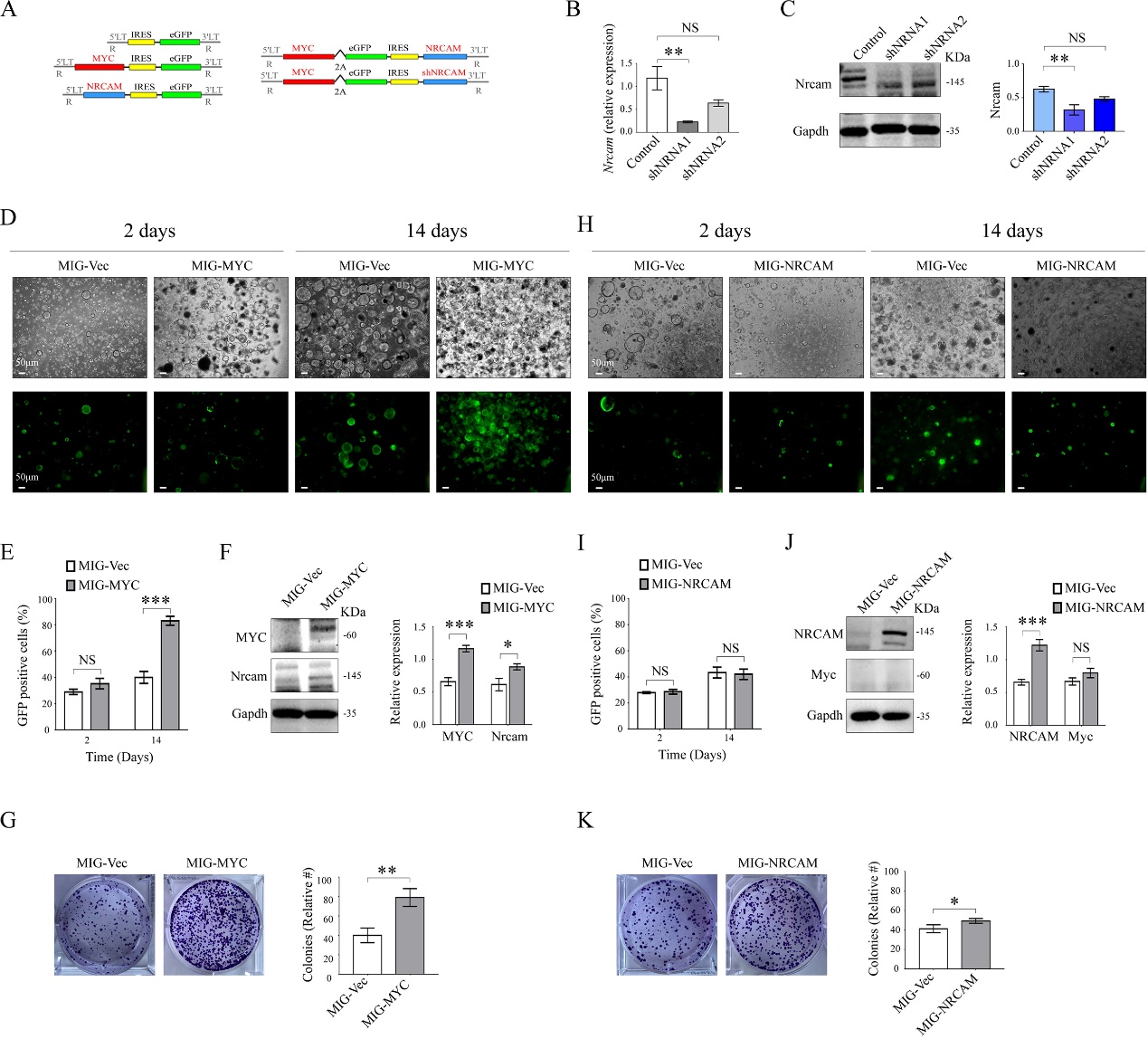
**

**Fig. S4.** **Regulation of NRCAM in mouse LCSCs.**

**(A)** MIG-Vec, MIG-MYC, MIG-NRCAM, MIG-MYC-NRCAM, and MIG-MYC-shNRCAM plasmid structures. **(B)** qPCR and **(C)** western blotting for shRNA-mediated NRCAM inhibition, shRNA1 had a good inhibitory effect on NRCAM and was used in subsequent experiments. **(D, E)** The GFP positive rate of MIG-Vec and MIG-MYC organoids two and 14 days after transduction. **(F)** MYC and Nrcam levels in MIG-Vec and MIG-MYC organoids. **(G)** Plate clone formation assay for MIG-Vec and MIG-MYC organoids. **(H, I)** GFP positive rate of MIG-Vec and MIG-NRCAM organoids two and 14 days after transduction. **(J)** NRCAM and Myc levels in MIG-Vec and MIG-NRCAM organoids. **(K)** plate clone formation assay. The data was displayed using the mean ± SD where applicable. P=<0.05 (*), P=<0.01 (**), P=<0.001 (***).

**
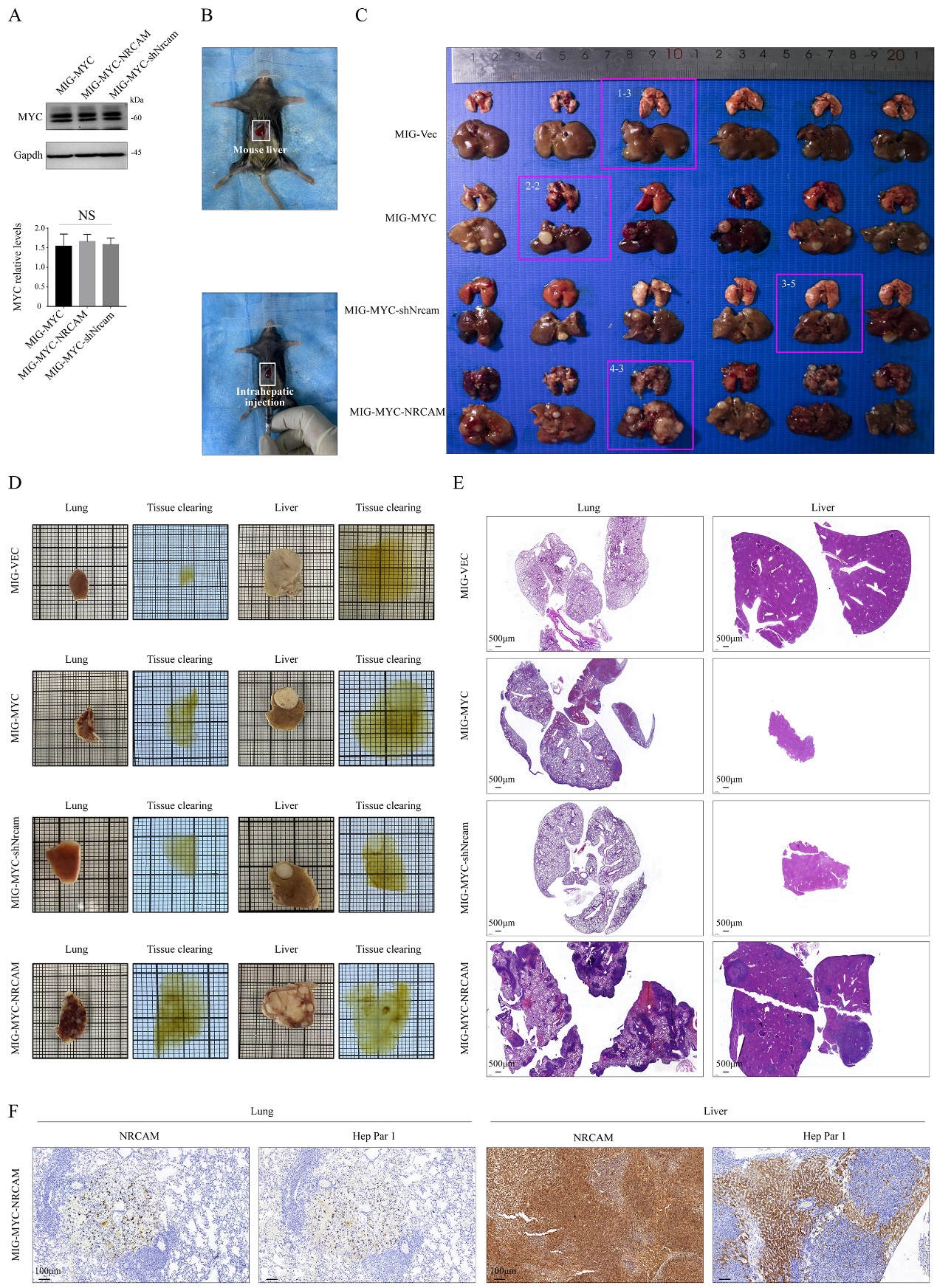
**

**Fig. S5. Identification of HCC metastasis in the murine oncogene-driven HCC allograft model.**

**(A)** MYC levels in MIG-MYC, MIG-MYC-NRCAM, and MIG-MYC-shNRCAM organoids. **(B)** Intrahepatic injection of MIG-Vec, MIG-MYC, MIG-MYC-NRCAM, and MIG-MYC-shNRCAM organoids into irradiation-induced transient immune deficient C57BL/6 mice. **(C)** Mouse lungs and livers 28 days after allografting for the MIG-Vec, MIG-MYC, MIG-MYC-shNRCAM, and MIG-MYC-NRCAM groups. **(D)** Tissue clearing and image analysis for lung and liver from the MIG-Vec (Mouse: 1-3), MIG-MYC (Mouse: 2-2), MIG-MYC-shNrcam (Mouse: 3-5), and MIG-MYC-NRCAM (Mouse: 4-3) group mice. **(E)** HE staining for the MIG-Vec (Mouse: 1-3), MIG-MYC (Mouse: 2-2), MIG-MYC-shNrcam (Mouse: 3-5) and MIG-MYC-NRCAM (Mouse: 4-3) groups. **(F)** IHC staining for NRCAM and Hep Par 1 in the lung and liver from MIG-MYC-NRCAM (Mouse: 4-3) group mice. The data was represented using the mean ± SD where applicable, P=>0.05 (NS).

**
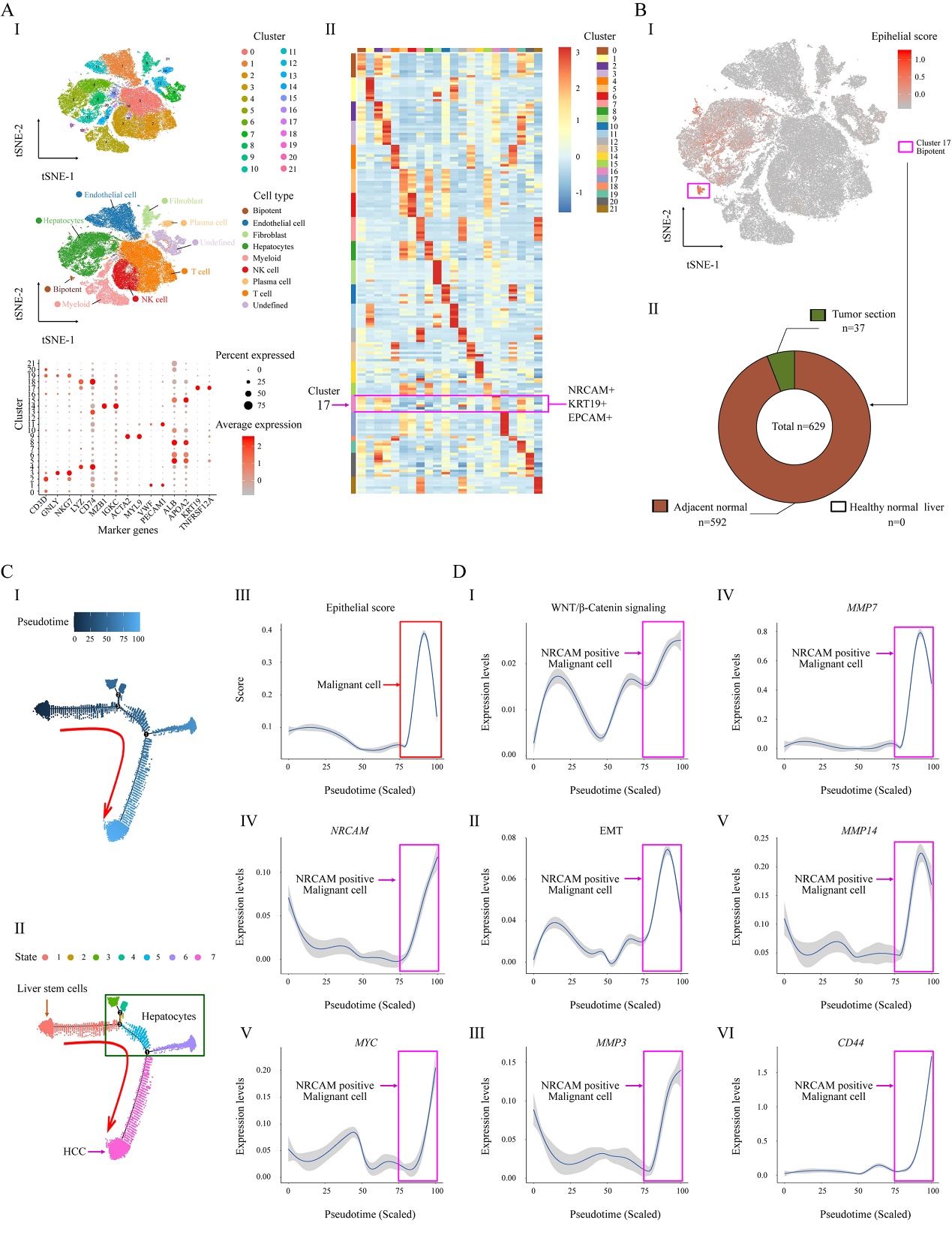
**

**Fig. S6. SRP278381 GEO database single-cell profile and evolution trajectory.**

**(A)** tSNE displaying the 21 main cell clusters and cell types, a differential gene expression analysis was used to identify cluster-specific markers. **(B)** *NRCAM* is predominately expressed in cluster 17, a group of bi-potent stem cells (KRT19+ and EPCAM+). **(C)** tSNE displaying malignant cells (red, identified using epithelial score), NRCAM+ bi-potent stem cells were malignant, and could be defined as LCSCs. NRCAM+ bi-potent stem cell distribution (Cluster 17) was mostly in adjacent normal tissue (adjacent normal: 592 from 629). **(D)** Hepatocyte trajectory analysis (arrow displays potential evolutionary direction): the potential trajectory of all hepatocytes, the color scale represents pseudotime; seven distinct cell states (colored by state 1-7) were identified across the potential hepatocyte trajectory. Liver stem cells were present in state 1, mature hepatocytes in state 2-6, and HCC cells in state 7. **(E)** Epithelial score genes, *NRCAM,* and *MYC* expression in pseudotime. **(F)** WNT/β-Catenin signaling activity, EMT activity, *MMP3,* *MMP7, MMP14,* and *CD44* expression in pseudotime.

**
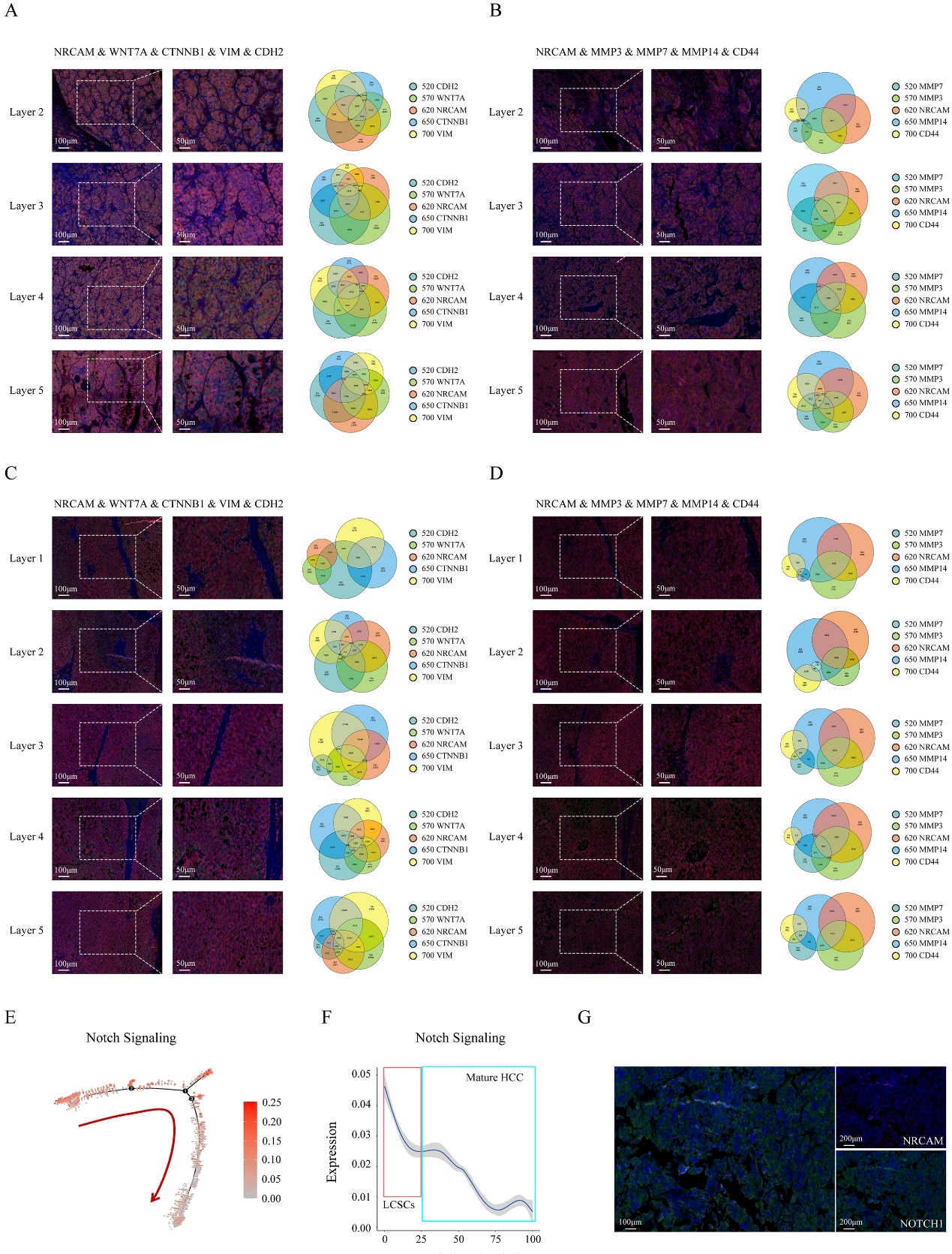
**

**Fig. S7 Identification of the key pathway associated with NRCAM activation in metastatic HCC.**

**(A)** Multiplex immunofluorescent (mIF) staining for NRCAM, Wnt/β-catenin signaling, and EMT in layers 2-5. Venn diagram displaying NRCAM, Wnt/β-catenin signaling, and EMT co-activation in HCC cells. **(B)** mIF staining for NRCAM, MMP3, MMP7, MMP14 and CD44. Venn diagram displaying MMP3, MMP7, MMP14, and CD44 co-activation in HCC cells. **(C)** mIF staining for NRCAM, Wnt/β-catenin signaling, and EMT in layer 1-5. Venn diagram displaying NRCAM, Wnt/β-catenin signaling, and EMT co-activation in HCC cells. **(D)** mIF staining for NRCAM, MMP3, MMP7, MMP14 and CD44. Venn diagram displaying MMP3, MMP7, MMP14, and CD44 co-activation in HCC cells. **(E)** Notch signaling activity on the trajectory (color scale representing activity level). **(F)** Notch signaling activity changes in pseudotime. **(G)** mIF staining for NRCAM and NOTCH1 in metastatic HCC tumor tissue demonstrates few cells with NRCAM and NOTCH1 co-activation.

**
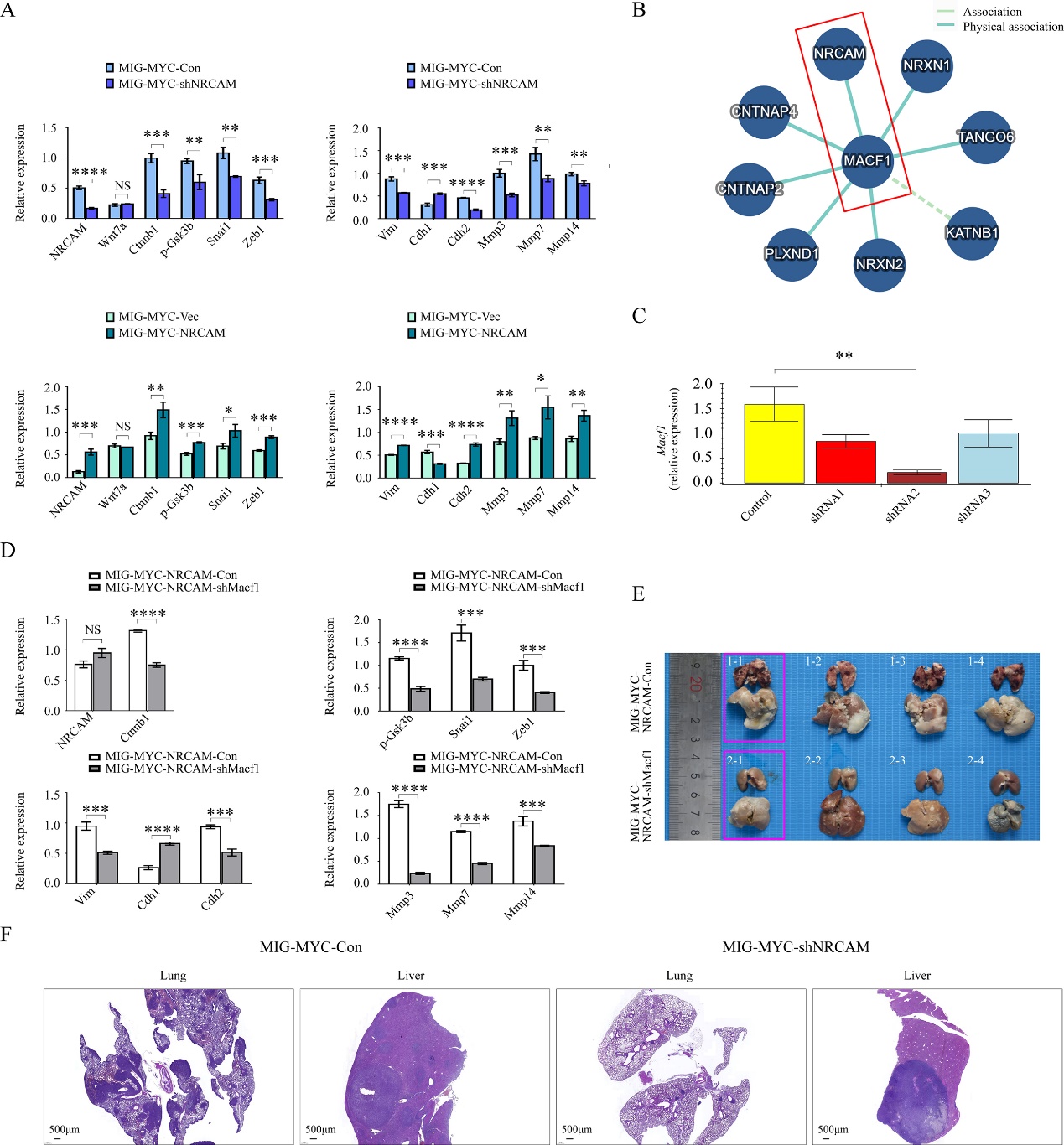
**

**Fig. S8. NRCAM, key factors in MACF1 mediated β-catenin signaling pathway, EMT and MMPs expression in LCSCs.**

**(A)** NRCAM, Wnt7a, Ctnnb1, p-Gsk3b, Snai1, Zeb1, Vim, Cdh1, Cdh2, Mmp3, Mmp7 and Mmp14 levels. **(B)** Interaction Network analysis of NRCAM and MACF1 from IntAct (<https://www.ebi.ac.uk/intact/home>). **(C)** shRNA inhibition of MACF1. The shRNA2 had a good inhibitory effect on MACF1 and was used in subsequent experiments. **(D)** NRCAM, Ctnnb1, p-Gsk3b, Snai1, Zeb1, Vim, Cdh1, Cdh2, Mmp3, Mmp7 and Mmp14 levels. **(E)** Mouse lungs and livers 28 days after allografting for the MIG-MYC-NRCAM-Con and MIG-MYC-NRCAM-shMacf1 groups. **(F)** HE staining for the MIG-MYC-NRCAM-Con (Mouse: 1-1) and MIG-MYC-NRCAM-shMacf1 (Mouse: 2-1) groups. Where applicable, the data was represented using mean ± SD (n=3). P=<0.05 (*), P=<0.01 (**), P=<0.001 (***), P=<0.0001 (****).

## Supplementary tables

**Table S1. Clinicopathological information for the two PDX HCC models**

| **Index** | **PDX1** | **PDX2** |
| --- | --- | --- |
| Gender | Male | Male |
| HBV infection | Yes | No |
| BCLC stages | A | A |
| HBV DNA | 2.31E+05 | N/A |
| AFP (ng/ml) | >1210 | 11.32 |
| PIVKA-II (mAU/ml) | 3034.00 | 2176.00 |
| MVI | no | Yes |
| Liver cirrhosis | Yes | Yes |
| Metastasis | No recurrence or metastasis two years post-surgery | Lung metastasis six months after surgery. |

MVI: microvascular invasion**Table S2. Clinicopathological information for five patients with HCC metastasis**

| **Index** | **HCC1** | **HCC2** | **HCC3** | **HCC4** | **HCC5** |
| --- | --- | --- | --- | --- | --- |
| Gender | Female | Male | Female | Male | Male |
| HBV infection | Yes | Yes | Yes | No | Yes |
| BCLC stages | A | A | B | B | B |
| HBV DNA | 1.96E+03 | 1.94E+03 | Undetectable | - | 3.44E+02 |
| AFP (ng/ml) | >1210 | 275.00 | 2.61 | 17.00 | >1210 |
| PIVKA-II (mAU/ml) | 3034.00 | 26251.00 | 81.00 | 92.00 | 333.00 |
| MVI | Yes | Yes | Yes | Yes | Yes |
| Liver cirrhosis | Yes | Yes | Yes | Yes | Yes |
| Serum NRCAM (pg/ml) | 3914.66 | 2473.08 | 1888.72 | 2355.74 | 2784.11 |

MVI: microvascular invasion

**Table S3. shRNAs targeting Nrcam and Macf1**

| **Genes** | **shRNA** |
| --- | --- |
| Nrcam-shRNA1-F | TGCTGTAAAGGTCTCCATTTAGGCCTGTTTTGGCCACTGACTGACAGGCCTAAGGAGACCTTTA |
| Nrcam-shRNA1-R | CCTGTAAAGGTCTCCTTAGGCCTGTCAGTCAGTGGCCAAAACAGGCCTAAATGGAGACCTTTAC |
| Nrcam-shRNA2-F | TGCTGTAGCAAAGCAGGCCTGTTTGCGTTTTGGCCACTGACTGACGCAAACAGCTGCTTTGCTA |
| Nrcam-shRNA2-R | CCTGTAGCAAAGCAGCTGTTTGCGTCAGTCAGTGGCCAAAACGCAAACAGGCCTGCTTTGCTAC |
| Macf1-shRNA1-F | TGCTGTAAAGATACCACCATCCTGTAGTTTTGGCCACTGACTGACTACAGGATTGGTATCTTTA |
| Macf1-shRNA1-R | CATTTCTATGGTGGTAGGACATCAAAACCGGTGACTGACTGATGTCCTAACCATAGAAATGTCC |
| Macf1-shRNA2-F | TGCTGTGAAGAAGCTTTCTCATCTTGGTTTTGGCCACTGACTGACCAAGATGAAAGCTTCTTCA |
| Macf1-shRNA2-R | CACTTCTTCGAAAGAGTAGAACCAAAACCGGTGACTGACTGGTTCTACTTTCGAAGAAGTGTCC |
| Macf1-shRNA3-F | TGCTGTATTCTTGAAGCTTTCCTGGAGTTTTGGCCACTGACTGACTCCAGGAACTTCAAGAATA |
| Macf1-shRNA3-R | CATAAGAACTTCGAAAGGACCTCAAAACCGGTGACTGACTGAGGTCCTTGAAGTTCTTATGTCC |

**Table S4.** **The qPCR primer sequences**

| **Genes** | **PCR primer sequences** |
| --- | --- |
| NRCAM-F (h) | GCTGGTATTCTTCCACCTGATG |
| NRCAM-R (h) | TTGCTGCCTGCTACACCATA |
| Nrcam-F (m) | GGAAGTCGAACACCTTCAGAC |
| Nrcam-R (m) | \| AGTTCATTGCGTTGACAGGAG \| \| --- \| |
| Macf1-F (m) | AACCTAGAAAGGGATCAGGTGG |
| Macf1-R (m) | GCTTGTTGGAGAGCTGCTCAA |
| B-Actin-F (m) | ACTATTGGCAACGAGCGGTTC |
| B-Actin-R (m) | ACGGATGTCAACGTCACACTTC |
| GAPDH-F | AGGAGCGAGATCCCTCCAAAATCAAGT |
| GAPDH-R | TGAGTCCTTCCACGATACCAAAGTTGT |

(h), human; (m), mouse

**Table S5. Antibodies**

| **Antibodies** | **Cat. No.** | **Companies** |
| --- | --- | --- |
| NRCAM | ab191814 | Abcam |
| NRCAM | ab24344 | Abcam |
| NOTCH1 | ab52627 | Abcam |
| MYC | ab32072 | Abcam |
| ALB | ab207327 | Abcam |
| KRT19 | ab76539 | Abcam |
| AFP | ab284388 | Abcam |
| MACF1 | ab117418 | Abcam |
| ARG1 | GT218 | Gene Tech |
| HepPar1 | GM715 | Gene Tech |
| WNT7A | 3113S | CST |
| VIM | 5741S | CST |
| CDH1 | 3195 | CST |
| CDH2 | 13116 | CST |
| CTNNB1 | 8480 | CST |
| p-GSK3B | 9323 | CST |
| SNAI1 | 3879 | CST |
| ZEB1 | 3396 | CST |
| MMP3 | ET1705-98 | HUABIO |
| MMP7 | HA500305 | HUABIO |
| MMP14 | ET1606-48 | HUABIO |
| GAPDH | HRP-60004 | Proteintech |
| Anti-rabbit IgG, HRP-linked Antibody | 7074S | CST |
| Goat anti-mouse IgG (H+L), HRP conjugate | SA00001-1 | Proteintech |

**Table S6. NrCAM mRNA in TCGA liver cancer and normal tissues.**

| **Sample types** | **N** | **Median** | **Upper quartile** | **Lower quartile** | **P** |
| --- | --- | --- | --- | --- | --- |
| HCC | 371 | 0.41 | 1.20 | 0.08 | <0.001 |
| Normal | 50 | 0.14 | 0.32 | 0.09 |  |

**Table S7. HCC diagnostic ROCs for AFP, PIVKA-II and NRCAM**

| **Factors** | **AUC (95% CI)** | **P value** | **Sig** |
| --- | --- | --- | --- |
| AFP | 0.86 (0.79-0.92) | <0.001 | *** |
| PIVKA-II | 0.86 (0.79-0.94) | <0.001 | *** |
| NRCAM | 0.84 (0.77-0.92) | <0.001 | *** |
| AFP & NRCAM | 0.90 (0.85-0.96) | <0.001 | *** |
| AFP & PIVKA-II | 0.90 (0.84-0.96) | <0.001 | *** |
| PIVKAII & NRCAM | 0.86 (0.79-0.93) | <0.001 | *** |
| AFP & PIVKAII & NRCAM | 0.91 (0.86-0.96) | <0.001 | *** |

Significance (Sig): P<0.001 (***).

**Table S8. Univariate and multivariate logistic analysis in HCC diagnosis**

| **Clinic Characteristics** | **Univariate** | | | **multivariate** | | |  |
| --- | --- | --- | --- | --- | --- | --- | --- |
|  | **Odds ratio (95% CI)** | **P value** | **Odds ratio (95% CI)** | | **P value** | **Sig** | |
| Age (years) | 1.01 (0.97-1.05) | 0.510 |  | |  |  | |
| Gender | 2.26 (0.92-5.52) | 0.039 | 0.83 (0.16-4.21) | | 0.820 |  | |
| Etiology | | | | | | |  |
| Unknown etiology | Reference |  |  | |  |  | |
| HBV | 9.72 (3.55-26.65) | <0.001 | 9.42 (2.65-33.50) | | 0.001 | ** | |
| HCV | 1.79 (0.11-8.33) | 0.443 |  | |  |  | |
| NAFLD | 1.56 (0.26-9.32) | 0.629 |  | |  |  | |
| ALD | 0.32 (0.02-4.26) | 0.354 |  | |  |  | |
| AIH | 2.53 (0.01-4.64) | 0.863 |  | |  |  | |
| Detectable HBV DNA viral load | 5.81 (2.02-16.69) | 0.335 |  | |  |  | |
| Liver cirrhosis | 8.21 (2.84-23.70) | <0.001 | 3.22 (0.60-17.39) | | 0.173 |  | |
| Platelets (103/mm3) | 0.99 (0.98-1.00) | 0.048 | 1.01 (0.99-1.02) | | 0.354 |  | |
| ALT (U/L) | 1.04 (1.01-1.08) | 0.006 | 1.03 (0.99-1.07) | | 0.121 |  | |
| AST (U/L) | 1.06 (1.02-1.11) | 0.004 | 0.98 (0.90-1.07) | | 0.703 |  | |
| Albumin (g/L) | 0.99 (0.90-1.08) | 0.705 |  | |  |  | |
| PT (s) | 2.58 (1.40-4.75) | 0.071 |  | |  |  | |
| Birrirubin (umol/L) | 0.99 (0.98-1.00) | 0.124 |  | |  |  | |
| AFP (ng/ml) | 1.04 (1.00-1.08) | 0.039 | 1.03 (0.99-1.06) | | 0.136 |  | |
| PIVKA-II (mAU/ml) | 1.00 (1.00-1.00) | 0.086 |  | |  |  | |
| NRCAM (pg/ml) | 1.00 (1.00-1.00) | <0.001 | 1.00 (1.00-1.00) | | 0.002 | ** | |

Significant (Sig): P<0.01 (**), P<0.001 (***). NAFLD, non-alcoholic fatty liver disease; ALD, Alcoholic liver disease; AIH, Autoimmune Hepatitis.

**Table S9. Pulmonary metastasis associated with different LCSCs.**

| **Groups** | **Tumors in lung** | **Largest tumor (mm^3^)** | **Tumor volume/Lung volume (%)** |
| --- | --- | --- | --- |
| MIG-Vec | 0 | 0 | 0 |
| MIG-MYC | 190 | 1.37 | 1.45 |
| MIG-MYC-shNrcam | 0 | 0 | 0 |
|  |  |  |  |
| MIG-MYC-NRCAM | 275 | 7.77 | 16.28 |
|  |  |  |  |

**Table S10. Intrahepatic metastasis associated with different LCSCs.**

| **Groups** | **Tumors in lung** | **Largest tumor (mm^3^)** | **Tumor volume/liver volume (%)** |
| --- | --- | --- | --- |
| MIG-Vec | 0 | 0 | 0 |
| MIG-MYC | 130 | 107.23 | 21.72 |
| MIG-MYC-shNrcam | 1 | 27.33 | 4.36 |
|  |  |  |  |
| MIG-MYC-NRCAM | 487 | 101.57 | 54.35 |
|  |  |  |  |

## Supplementary References

1. Chen S, Zhou Y, Chen Y, Gu J. fastp: an ultra-fast all-in-one FASTQ preprocessor. Bioinformatics. 2018;34(17):i884-i90.

2. Zheng GX, Terry JM, Belgrader P, Ryvkin P, Bent ZW, Wilson R, et al. Massively parallel digital transcriptional profiling of single cells. Nat Commun. 2017;8:14049.

3. Dobin A, Davis CA, Schlesinger F, Drenkow J, Zaleski C, Jha S, et al. STAR: ultrafast universal RNA-seq aligner. Bioinformatics. 2013;29(1):15-21.

4. Jaitin DA, Kenigsberg E, Keren-Shaul H, Elefant N, Paul F, Zaretsky I, et al. Massively parallel single-cell RNA-seq for marker-free decomposition of tissues into cell types. Science. 2014;343(6172):776-9.

5. Klein AM, Mazutis L, Akartuna I, Tallapragada N, Veres A, Li V, et al. Droplet barcoding for single-cell transcriptomics applied to embryonic stem cells. Cell. 2015;161(5):1187-201.
